# Targeting pleckstrin-2/Akt signaling reduces proliferation in myeloproliferative neoplasm models

**DOI:** 10.1172/JCI159638

**Published:** 2023-03-15

**Authors:** Xu Han, Yang Mei, Rama K. Mishra, Honghao Bi, Atul D. Jain, Gary E. Schiltz, Baobing Zhao, Madina Sukhanova, Pan Wang, Arabela A. Grigorescu, Patricia C. Weber, John J. Piwinski, Miguel A. Prado, Joao A. Paulo, Len Stephens, Karen E. Anderson, Charles S. Abrams, Jing Yang, Peng Ji

**Affiliations:** 1 Department of Pathology, Feinberg School of Medicine,; 2 Robert H. Lurie Comprehensive Cancer Center,; 3 Department of Biochemistry and Molecular Genetics, Feinberg School of Medicine,; 4 Department of Chemistry, and; 5 Department of Pharmacology, Feinberg School of Medicine, Northwestern University, Chicago, Illinois, USA.; 6 Department of Molecular Biosciences, Weinberg College of Arts & Sciences, Northwestern University, Evanston, Illinois, USA.; 7 Harrington Discovery Institute, Cleveland, Ohio, USA.; 8 Department of Cell Biology, Harvard Medical School, Boston, Massachusetts, USA.; 9 Signaling Programme, The Babraham Institute, Cambridge, United Kingdom.; 10 Department of Medicine, University of Pennsylvania, Philadelphia, Pennsylvania, USA.

**Keywords:** Hematology, Oncology, Bone marrow, Cancer, Drug screens

## Abstract

Myeloproliferative neoplasms (MPNs) are characterized by the activated JAK2/STAT pathway. Pleckstrin-2 (Plek2) is a downstream target of the JAK2/STAT5 pathway and is overexpressed in patients with MPNs. We previously revealed that Plek2 plays critical roles in the pathogenesis of JAK2-mutated MPNs. The nonessential roles of Plek2 under physiologic conditions make it an ideal target for MPN therapy. Here, we identified first-in-class Plek2 inhibitors through an in silico high-throughput screening approach and cell-based assays, followed by the synthesis of analogs. Plek2-specific small-molecule inhibitors showed potent inhibitory effects on cell proliferation. Mechanistically, Plek2 interacts with and enhances the activity of Akt through the recruitment of downstream effector proteins. The Plek2-signaling complex also includes Hsp72, which protects Akt from degradation. These functions were blocked by Plek2 inhibitors via their direct binding to the Plek2 dishevelled, Egl-10 and pleckstrin (DEP) domain. The role of Plek2 in activating Akt signaling was further confirmed in vivo using a hematopoietic-specific *Pten*-knockout mouse model. We next tested Plek2 inhibitors alone or in combination with an Akt inhibitor in various MPN mouse models, which showed significant therapeutic efficacies similar to that seen with the genetic depletion of Plek2. The Plek2 inhibitor was also effective in reducing proliferation of CD34-positive cells from MPN patients. Our studies reveal a Plek2/Akt complex that drives cell proliferation and can be targeted by a class of antiproliferative compounds for MPN therapy.

## Graphical abstract

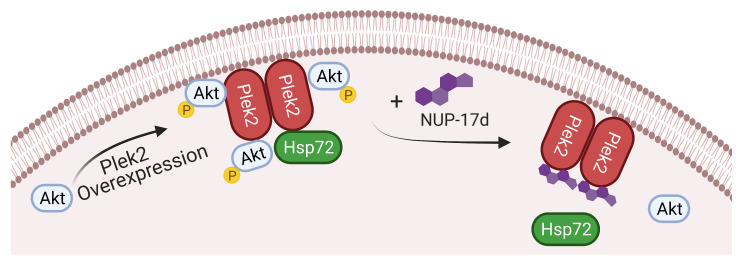

## Introduction

Philadelphia chromosome–negative (Ph-negative) myeloproliferative neoplasms (MPNs) are a group of BM diseases featuring excessive production of myeloid cells and increased risk of evolving to acute myeloid leukemia. JAK2^V617F^ mutation is the leading cause of Ph-negative MPNs that include polycythemia vera (PV), essential thrombocythemia (ET), and primary myelofibrosis (PMF) (1–5). Unlike with many effective therapies for Ph-induced MPNs, developing effective therapies for Ph-negative MPNs is in its nascence. The FDA-approved JAK inhibitor ruxolitinib reduces spleen size and blood cell counts in MPN patients (6–8). However, it is associated with marked long-term side effects, including anemia and thrombocytopenia, due to the essential role of JAK2 in hematopoiesis. Therefore, new therapeutic strategies are needed for treating Ph-negative MPNs, such as compounds that target JAK/STAT effectors that may cause fewer side effects.

Pleckstrin-2 (Plek2) is a membrane protein and paralog of Plek1. Plek2 is widely expressed and involved in actin rearrangement (9). Overexpression of Plek2 induces lamellipodia and peripheral ruffle formation (10). We previously showed that Plek2 is important in the regulation of actin cytoskeleton, cell differentiation, and apoptosis in terminal erythroid differentiation (11). Subsequent studies revealed that Plek2 is a downstream target of the JAK2/STAT5 pathway and overexpressed in JAK2^V617F^-positive patients with MPNs. In addition to the erythroid lineage, Plek2 is also upregulated in the myeloid and megakaryocytic lineages induced by JAK2^V617F^ mutation. The critical role of Plek2 in the pathogenesis of MPNs was manifested by a JAK2^V617F^-knockin mouse model in which loss of Plek2 largely rescued the lethality of the JAK2 mutant mice (12). Plek2 deficiency not only ameliorated myeloproliferative phenotypes in the JAK2^V617F^ mutant mice; it also markedly reduced thrombosis in many organ systems. Notably, Plek2-KO mice exhibited only minor age-related anemia without other severe phenotypes, which indicates that Plek2’s proproliferative function is tumor specific (12).

Plek2 is not only highly expressed in the hematopoietic cells; recent studies demonstrated that it is also overexpressed in many solid tumors and associated with worse prognosis (13–16). Specifically, Plek2 gene upregulation was predicted in 2 independent studies to result in a shorter progression-free survival and poor prognosis in lung adenocarcinoma (14, 15). Plek2’s high expression was associated with poor overall survival in gastric cancer (17). The mechanisms of Plek2 in tumorigenesis can be tissue specific, as Plek2 is reported to promote the invasion and metastasis of gallbladder cancer through binding and activation of EGFR (13). Plek2 could also be negatively regulated by miR-873. Reduced expression of miR-873 in pancreatic cancer stem cell led to upregulation of Plek2 and activation of the PI3K pathway (16). The possible role of Plek2 in the PI3K pathway was further indicated through bioinformatic analyses in osteosarcoma (18). However, the mechanisms of Plek2 in driving cell proliferation through PI3K signaling are unknown.

Plek2 contains 2 pleckstrin homology (PH) domains on the amino and carboxyl termini of the protein, which flank a dishevelled, Egl-10, and pleckstrin (DEP) domain. Previous loss-of-function studies showed that deletion of the DEP domain induced the most significant functional defects of Plek2 (10, 19). In this study, we aimed to reveal the mechanism of Plek2 in MPN pathogenesis and identify small-molecule compounds that target Plek2 and block its functions in MPNs. To this end, we first took an in silico approach to screening small molecules that bind to the DEP domain of Plek2 and inhibit its function in driving proliferation. These inhibitors were then used to explore the roles of Plek2 in MPNs. We found that Plek2 functioned as a regulatory protein to directly interact with Akt and recruit PI3K effector proteins as well as heat shock protein Hsp72, which led to the activation and stabilization of Akt. The functions of Plek2 in Akt activation were further demonstrated using a *Pten* hematopoietic-specific KO mouse model. We further revealed the therapeutic potentials of the Plek2 inhibitors in various MPN models. These studies establish Plek2’s critical role in driving cell proliferation when overexpressed. Thus, the small-molecule Plek2 inhibitors have important implications for treating MPNs and possibly other cancers with activated Akt signaling.

## Results

### Development of Plek2 small-molecule inhibitors that block cell proliferation.

To screen small molecules that bind to Plek2 and potentially inhibit its function, we chose to focus on the DEP domain instead of the PH domains that can be found in many proteins. The DEP domain of Plek2 is highly conserved among different species (Supplemental Figure 1A; supplemental material available online with this article; https://doi.org/10.1172/JCI159638DS1). As there are no reported crystal structures, we built a homology model of Plek2 using prime module (20) incorporated in the Schrödinger platform (https://www.schrodinger.com/products/prime), considering the primary amino acid sequence of the DEP domain as the query, and identified relevant template structures by homology search using BLAST and PSI-BLAST engines. The Plek2 model was then subjected to MolProbity validation, and the model scored 95% or more (21), indicating its suitability for carrying out further in silico screening. We then performed a virtual high-throughput screening (vHTS) using a 3-tier Glide platform implemented in Schrödinger suite (22). We screened approximately 100,000 drug-like small-molecule compounds for those that could bind to the Plek2 DEP domain. From this set, we identified 28 hit compounds that potentially bind to Plek2 (Supplemental Figure 1, B and C, and Supplemental Table 1).

**Figure 1:**
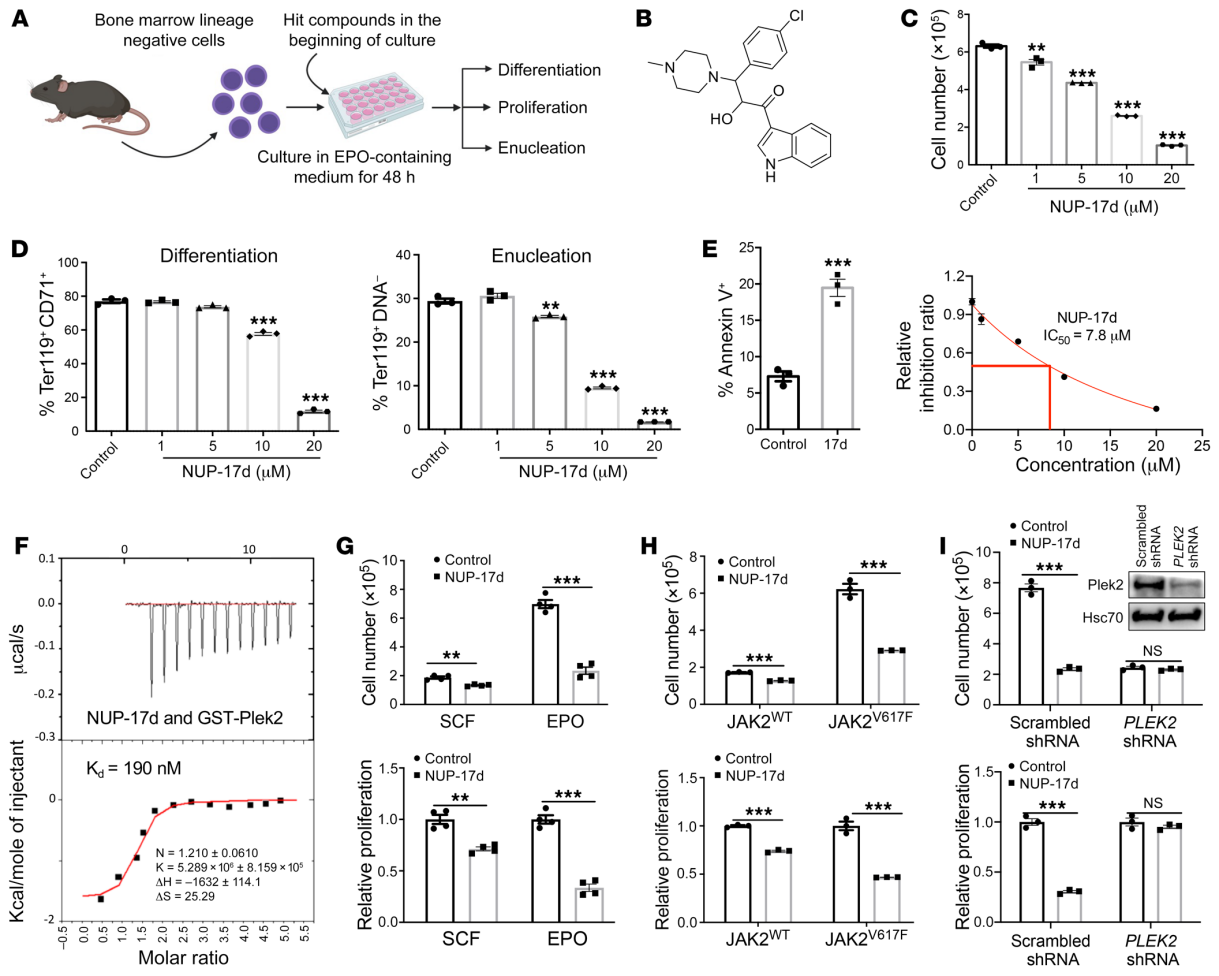
Development of Plek2 small-molecule inhibitors that block cell proliferation. (**A**) Schematics of HSPC in vitro culture system for the secondary screen of the hit compounds. Graph is generated using BioRender. (**B**) The chemical structure of NUP-17d. (**C**) BM lineage–negative cells were cultured in EPO-containing medium with different amounts of NUP-17d added at the beginning of culture. Cell proliferation was analyzed at 48 hours in culture. IC_50_ was calculated. (**D** and **E**) Same as **C** except cell differentiation, enucleation in **D**, and apoptosis in **E** were analyzed using flow cytometry. (**F**) ITC analyses demonstrate a direct interaction of NUP-17d with full-length Plek2. (**G**) 1 × 10^5^ BM lineage–negative cells were cultured with EPO medium as in **C** or medium containing stem cell factor (SCF). NUP-17d (10 μM) was added at the beginning of cell culture. Cell proliferation was analyzed at 48 hours in culture. (**H**) BM lineage–negative cells purified from the indicated mice were cultured in SCF medium as in **G**. NUP-17d (10 μM) was added at the beginning of cell culture. Cell proliferation was analyzed at 48 hours in culture. (**I**) BM lineage–negative cells transduced with Plek2 shRNA or control scrambled shRNA were cultured in EPO medium and treated with NUP-17d as in **G**. Cell proliferation was analyzed at 48 hours in culture. Western blotting assay demonstrates the downregulated Plek2 levels. Error bars represent SEM of the mean. Comparisons between 2 groups were evaluated with 2-tailed *t* test, and comparisons among multiple groups were evaluated with 1-way ANOVA. **P < 0.01; ***P < 0.001.

The hit compounds were subsequently screened using a mouse BM erythroid progenitor culture system in erythropoietin-containing (EPO-containing) medium (Figure 1A) (11, 23, 24). These cells underwent rapid proliferation with supraphysiological levels of EPO and closely mimicking accelerated erythropoiesis in MPNs. In these cells, Plek2 was also highly expressed, especially in the late stages of the culture (Supplemental Figure 1D). We tested each of the 28 hits in this system and found compound 17 (NUP-17) to have the most significant inhibitory effects on erythroid proliferation and enucleation (Supplemental Figure 1, E and F). The IC_50_ in blocking cell proliferation was 56 μM. NUP-17 also had a significant inhibitory effect on enucleation, but minimal effects on cell differentiation (Supplemental Figure 2A).

Based on similarity searching from the in silico screening, we further identified a series of analogs of NUP-17 and found that compound NUP-17d had significantly improved potency (Supplemental Figure 2, B and C, and Figure 1B). The IC_50_ of NUP-17d was 7.8 μM in the erythroid in vitro proliferation assay (Figure 1C). NUP-17d also significantly inhibited enucleation and differentiation (at higher dosage) of the cultured erythroid cells (Figure 1D) and induced apoptosis (Figure 1E). NUP-17d bound to Plek2 and Plek2-DEP with high affinity (*K_D_* = 190 and 305 nM, respectively) in an isothermal titration calorimetry (ITC) assay (Figure 1F and Supplemental Figure 2D). NUP-17d reduced cell proliferation more significantly when the BM lineage–negative cells were cultured in EPO-containing medium compared with those cultured in medium containing stem cell factor to maintain their progenitor status (Figure 1G). We also tested erythroblasts from JAK2^V617F^-knockin mice with NUP-17d, which showed that JAK2^V617F^-positive erythroblasts were more sensitive to the compound treatment (Figure 1H). The effect of NUP-17d was Plek2 specific, since no inhibitory effect was observed when Plek2 was acutely depleted by shRNA (11) (Figure 1I).

In addition, we also overexpressed Plek2 in Cos-7 cells, which induced prominent lamellipodia formation, as previously reported (9). NUP-17, and to a greater extent NUP-17d, dramatically reverted lamellipodia in Plek2-overexpressed cells (Supplemental Figure 2, E and F). We next constructed a biotin-conjugated NUP-17d (Supplemental Figure 3A) and confirmed NUP-17d binding to Plek2 and Plek2-DEP in in vitro binding assays (Supplemental Figure 3, B and C). Addition of free NUP-17d reduced the binding of biotin–NUP-17d to Plek2 (Supplemental Figure 3D). Cellular thermal shift assay (CETSA) further revealed specific interaction of NUP-17d with Plek2 in vivo (Supplemental Figure 3E). These studies establish NUP-17d as an effective Plek2 inhibitor.

To further characterize the specificity of the binding between NUP-17d and Plek2 DEP, we applied NUP-17d to the docking model and revealed lysine 156, serine 162, and arginine 194 to be critical for binding (Supplemental Figure 4A). Sequence alignment of the DEP domain in various proteins showed that these residues are not conserved and are highly specific to Plek2 (Supplemental Figure 4B). We next mutated these residues to asparagine, alanine, and glycine, respectively and obtained a recombinant Plek2 DEP 3M protein. An in vitro ITC experiment showed that NUP-17d completely lost its binding to DEP 3M (Supplemental Figure 4C). We further generated a full-length Plek2 3M construct and transduced it into Cos-7 cells. Compared with the WT Plek2, the Plek2 3M mutant failed to increase lamellipodia formation (Supplemental Figure 4, D and E). The mutant also failed to promote proliferation of HEL cells, a hematopoietic cell line harboring a JAK2 mutation (Supplemental Figure 4F). These studies indicate that the inhibitor-binding residues of Plek2 are critical for its function.

### Plek2 inhibitors block Plek2-mediated Akt activation.

**Figure 2:**
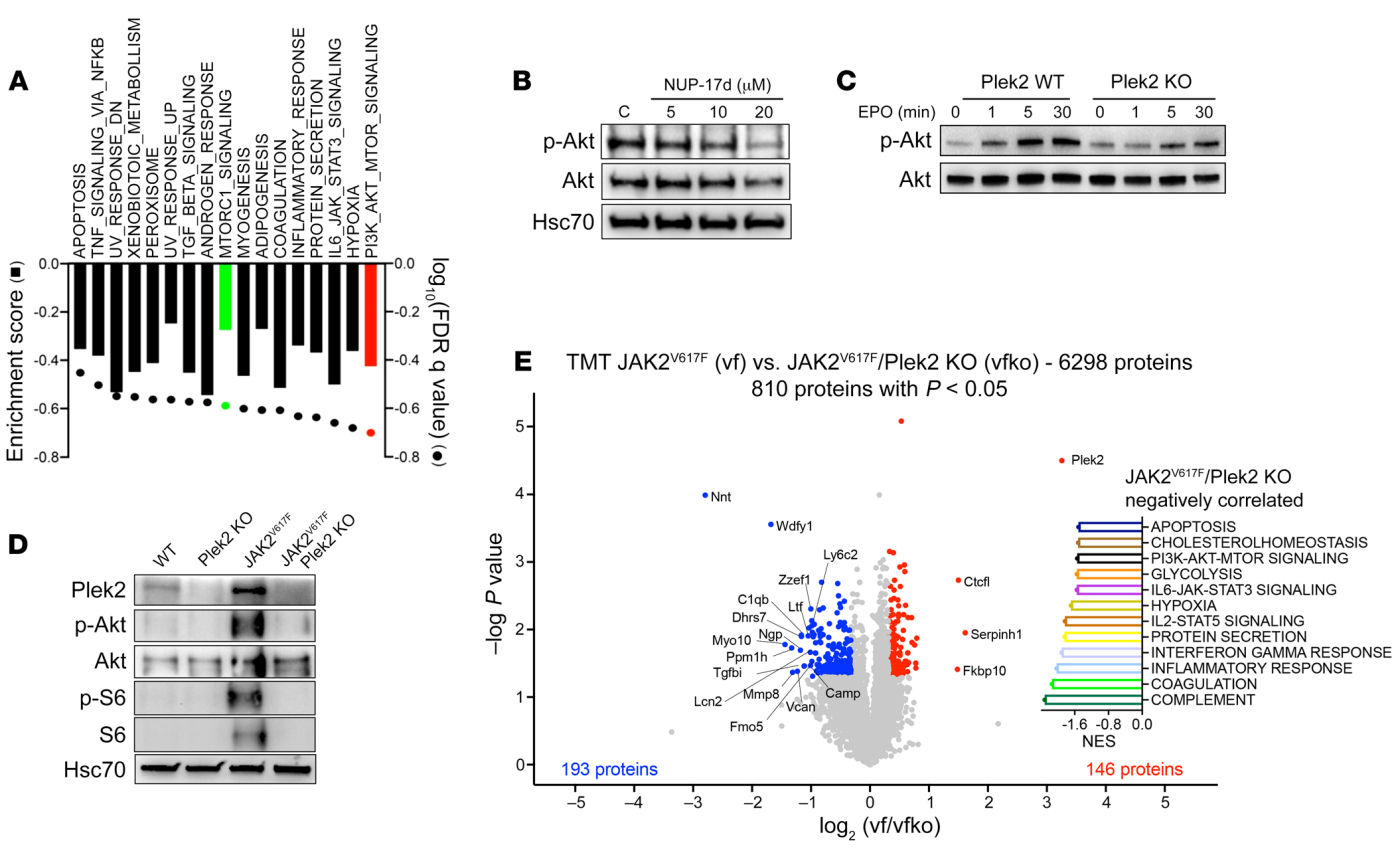
Plek2 is involved in the regulation of Akt signaling. (**A**) RNA-Seq analysis of BM lineage–negative cells cultured in EPO-containing medium with or without NUP-17d (10 μM) for 48 hours. Pathways enriched in NUP-17d–treated cells are shown. (**B**) BM lineage–negative cells were cultured in EPO medium for 24 hours. Cells were then treated with increasing concentrations of NUP-17d for 2 hours. Western blot assays of the indicated proteins were performed. (**C**) Western blot assays of p-Akt and total Akt in the starved BM TER119^+^ cells from Plek2 WT and KO mice followed by treatment with 2 units of EPO for the indicated durations. Total Akt was used for equal loading among the samples. (**D**) Western blot assays of the indicated proteins from total BM cells of the indicated mice at 3 months old. (**E**) Volcano plot of quantitative proteomic study. Proteins significantly up- (blue) or downregulated (red) in JAK2^V617F^-knockin/Plek2-KO TER119^+^ cells are presented. JAK2^V617F^/Plek2-KO negatively correlated pathways in GSEA analyses are shown on the right. Experiments were repeated 4 times, and data were obtained with combined individual analysis.

Plek2 is associated with membranes via its PH domains, and that association is dependent on D3 phosphoinositide upon the activation of PI3K (10). The mechanisms of Plek2 in driving cell proliferation remain unclear. We performed an RNA-Seq analysis in the cultured BM erythroblasts treated with NUP-17d. Compared with what occurred in the mock-treated cells, genes in several pathways, including PI3K/AKT/mTOR signaling and mTORC1 signaling, were enriched (Figure 2A), which was confirmed by gene set enrichment analyses (GSEAs) (Supplemental Figure 5A). Consistently, treatment of the cultured erythroid cells with NUP-17d significantly reduced the level of phosphorylated Akt (p-Akt). Interestingly, the protein level of Akt was also downregulated with a higher dose of the compound (Figure 2B). These data indicate that Plek2 could be involved in the activation of the Akt pathway. To test this hypothesis, we first compared the levels of p-Akt in TER119-positive erythroblasts from Plek2 WT and KO mice stimulated by EPO after starvation. Total Akt protein levels were equalized among samples to test the direct role of Plek2 on Akt phosphorylation. Plek2-deficient erythroblasts indeed exhibited delayed and attenuated increases of p-Akt upon EPO stimulation (Figure 2C). We previously reported that loss of Plek2 reverted myeloproliferative phenotypes in a JAK2^V617F^-knockin mouse model (12). The JAK2^V617F^ mutation is known to activate Akt, but the mechanism is incompletely understood. To understand whether Plek2 is involved in the activation of Akt in JAK2^V617F^-knockin mice, we purified total BM cells from WT, Plek2-KO, JAK2^V617F^-knockin, and JAK2^V617F^-knockin/Plek2-KO mice. As expected, loss of Plek2 significantly diminished the upregulated p-Akt, p-S6, and total S6 induced by JAK2^V617F^ (Figure 2D). The protein level of Akt remained unchanged due to possible compensatory effects in vivo. We next compared the proteome of these cells using a tandem mass tagging (TMT) mass spectrometry assay and found the downregulated PI3K/Akt pathway among others in cells from JAK2^V617F^-knockin/Plek2-KO mice when compared with those from JAK2^V617F^-knockin mice (Figure 2E).

### Plek2 forms a membrane-signaling complex to activate and upregulate Akt.

**Figure 3:**
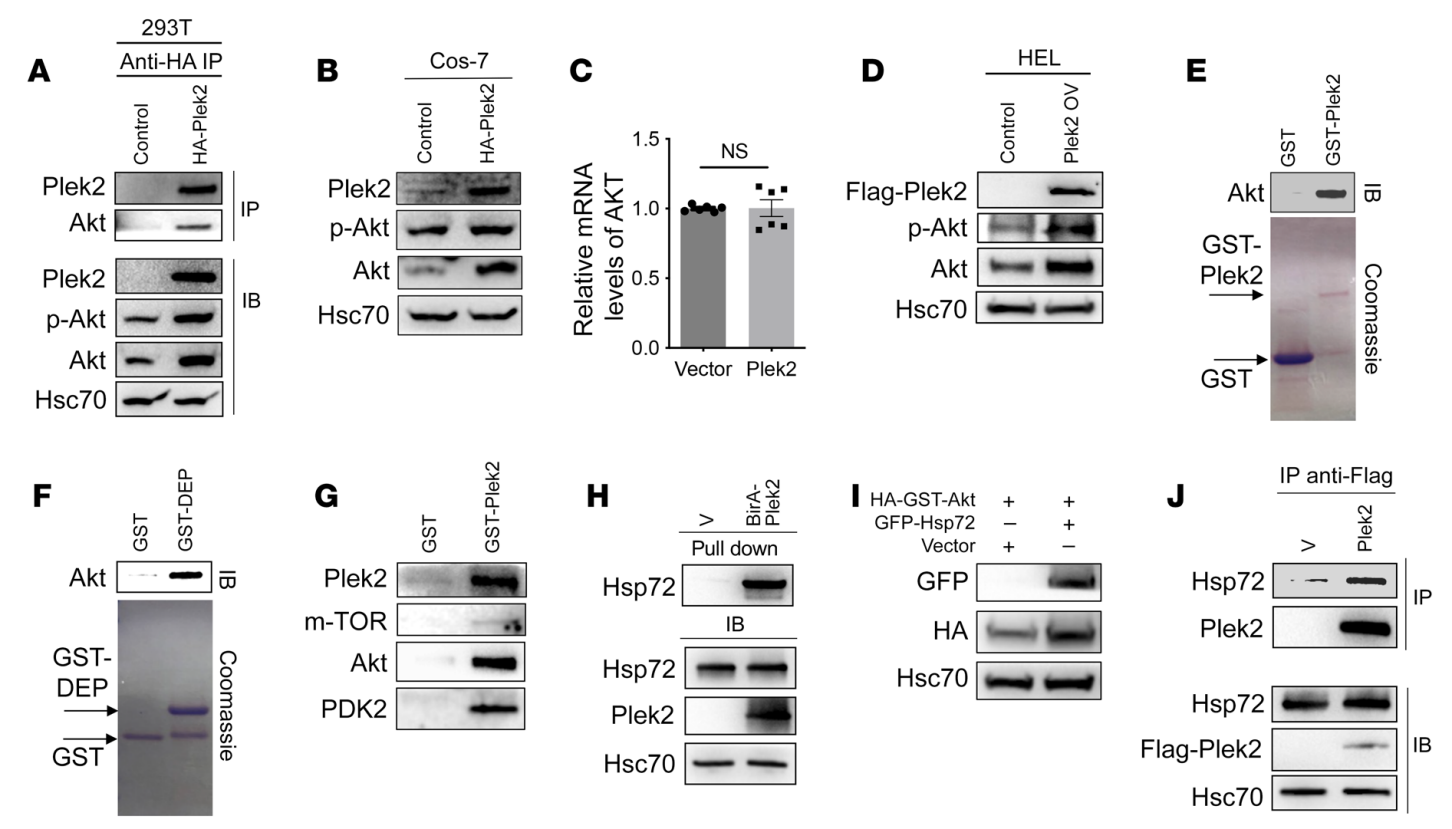
Plek2 activates Akt and protects Akt through Hsp72. (**A**) IP of anti-HA with cell lysate from 293T cells transfected with HA-Plek2, followed by Western blot assays of indicated proteins. (**B**) Western blotting assays of indicated proteins from Cos-7 cells transfected with HA-Plek2. (**C**) Quantitative PCR analysis of *AKT* from Cos-7 cells transfected with HA-Plek2. (**D**) Western blotting assays of indicated proteins from HEL cells transfected with Flag-Plek2. (**E**) GST pull-down assay using GST or GST-Plek2 with a recombinant Akt. Akt was detected by a Western blotting assay. GST and GST-Plek2 were revealed using Coomassie stain. (**F**) Same as **E**, except GST-Plek2 DEP was used. (**G**) GST pull-down assay of GST or GST-Plek2 incubated with cell lysis from 293T cells. Indicated proteins were detected by Western blotting. (**H**) Bead-conjugated streptavidin pull-down of 293T cells transfected with BirA-Plek2 and incubated with biotin (50 μM). A Western blotting assay of the indicated proteins was performed after SDS-PAGE. (**I**) Western blotting analyses of the indicated proteins in 293T cells transfected with GFP fusion Hsp72 and HA-GST-Akt. (**J**) IP of Flag tag with cell lysate from 293T cells transfected with Flag-Plek2 followed by a Western blotting assay of indicated proteins.

PI3K phosphorylates the 3-hydroxyl group of membrane phospholipid phosphatidylinositol 4,5-biphosphate (PI[4,5]P_2_) to generate the lipid signaling messenger PI(3,4,5)P_3_, which serves as a docking site on the membrane for the PH domain of Akt and partially activates Akt. PI(3,4,5)P_3_ can be metabolized to produce PI(3,4)P_2_, and recent evidence points to additional signaling roles for this lipid. Akt binds most strongly to PI(3,4)P_2_ among PI and phosphatidylinositol phosphates (PIPs) that are important messengers for signaling transduction (25). Plek2 also binds more strongly to PI(3,4)P_2_ (Supplemental Figure 5B). In certain cell types, under specific contexts, PI(3,4)P_2_ can accumulate to levels higher than that of PI(3,4,5)P_3_ (26). Here, we found that PI(3,4)P_2_ was significantly more abundant than PI(3,4,5)P_3_ in the BM erythroblasts, which was further increased upon stimulation of the cells with EPO (Supplemental Figure 5C). Based on these results, we reasoned that Plek2 could influence Akt activation through their membrane association to PI(3,4)P_2_. To explore the relationship of Plek2 and Akt, we first performed a coimmunoprecipitation (co-IP) assay and found interaction of Plek2 with Akt (Figure 3A). Domain-mapping studies revealed that the C-terminal catalytic and regulatory domains of Akt are involved in the binding to Plek2 (Supplemental Figure 5D). The protein levels of total Akt and p-Akt, but not the mRNA level of Akt, were also elevated when Plek2 was overexpressed in both 293T cells and Cos-7 cells (Figure 3, A–C). Overexpression of Plek2 also led to increased Akt and p-Akt levels in HEL cells (Figure 3D). The Plek2/Akt interaction was confirmed in vitro in that GST-tagged Plek2 or Plek2 DEP domain directly interacted with recombinant Akt (Figure 3, E and F). A GST pull-down assay revealed that PI3K/Akt effector proteins, such as mTOR and PDK2, were also recruited in the Plek2/Akt complex (Figure 3G). Together, these results indicate that Plek2 functions as a regulatory protein to recruit proteins in the Akt-signaling pathway to increase Akt protein level and activity.

Akt is known to be regulated by ubiquitination for its stability and activity. Its polyubiquitination by TRAF6 (27), NEDD4 (28), and Skp2 (29) was reported to enhance the kinase activity whereas Mul1 (30) downregulates Akt. We found that Plek2 overexpression increased the level of polyubiquitinated Akt (Supplemental Figure 5E), indicating that Plek2 upregulates Akt through pathways other than inhibiting its ubiquitination and proteasome degradation. Indeed, the increase in Akt polyubiquitination was through K63, which is known to be critical for Akt activation (Supplemental Figure 5, F and G). To uncover the mechanism of how Plek2 overexpression leads to increased Akt protein levels, we performed an affinity purification using biotin conjugation and streptavidin pull-down of the cell lysate from 293T cells expressing BirA-fusion Plek2. Besides Plek2, the most abundant protein that precipitated with streptavidin beads was Hsp72 (Supplemental Figure 5H and Supplemental Table 2), which was previously reported to stabilize Akt (31). Western blotting assays confirmed the presence of Hsp72 in the complex and its effect on the upregulation of Akt protein levels (Figure 3, H and I). A co-IP assay further demonstrated an interaction between Plek2 and Hsp72 (Figure 3J). These results demonstrate that Plek2 upregulates Akt through the recruitment of Hsp72.

### Plek2 inhibitors lead to Akt dissociation from the lipid/Plek2 complex and instability.

**Figure 4:**
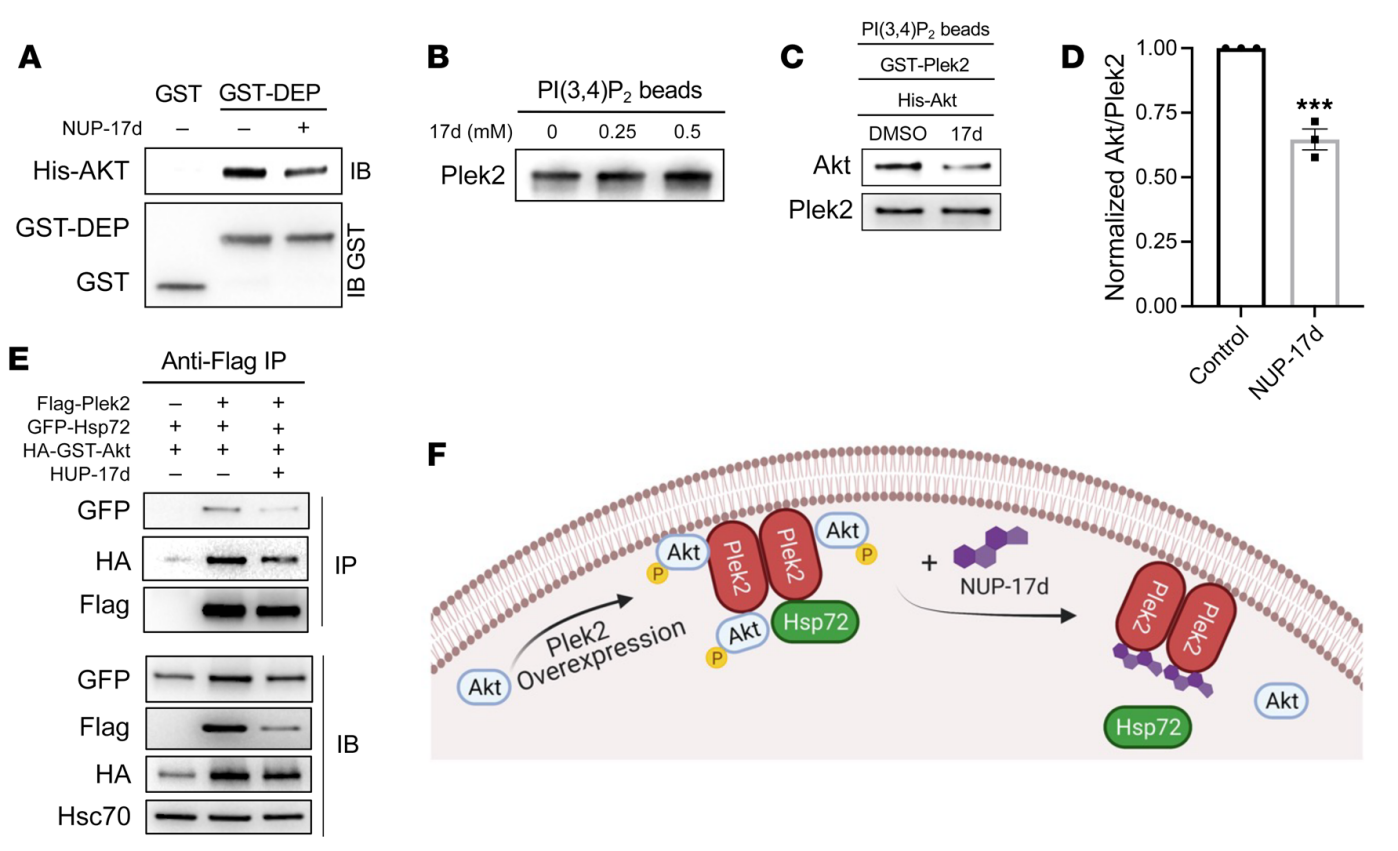
Plek2 inhibitors block Plek2-mediated Akt activation. (**A**) GST pull-down assay of GST-DEP and His-Akt in the presence or absence of 500 μM NUP-17d, followed by a Western blotting assay of Akt and GST. (**B**) Bead-conjugated PI(3,4)P_2_ incubated with recombinant Plek2 and treated with increasing concentrations of NUP-17d, followed by a Western blot of Plek2. (**C**) Bead-conjugated PI(3,4)P_2_ incubated with recombinant Akt and Plek2 was treated with or without 500 μM NUP-17d for 2 hours, followed by a Western blotting assay of the indicated proteins. (**D**) Quantification of the normalized Akt/Plek2 protein ratio in 3 independent experiments in **C**. ***P < 0.001. (**E**) IP of anti-Flag with cell lysate from 293T cells transfected with indicated constructs, followed by the treatment of 20 μM NUP-17d for 2 hours. Western blotting assays were then performed. (**F**) Schematic illustration of the mechanisms of Plek2 and Plek2 inhibitors. Graph was generated using BioRender.

With this knowledge, we next determined the mechanisms of function of Plek2 inhibitors. An ITC assay revealed no interaction between NUP-17d and Akt, further confirming the specificity of Plek2 inhibitors on Plek2 (Supplemental Figure 5I). Consistent with the targeting of the Plek2 DEP domain, NUP-17d reduced the interaction between Plek2 DEP and Akt (Figure 4A), but did not interfere with Plek2’s lipid affinity (Figure 4B). We then applied NUP-17d in the in vitro binding assay with the bead-conjugated PI(3,4)P_2_. NUP-17d significantly reduced lipid association of Akt in the presence of Plek2 (Figure 4, C and D). When NUP-17d was applied in cells, it reduced the interactions of both Akt and Hsp72 with Plek2. The increase in the level of Akt by Plek2 overexpression was also reduced (Figure 4E). Taken together, these data reveal that overexpression of Plek2 results in increased Akt ubiquitination, activation, and stability. Through binding of the DEP domain of Plek2, Plek2 inhibitors induce a confirmation change of Plek2 protein, which leads to Akt dissociation from the lipid/Plek2 complex and instability (Figure 4F).

### Plek2 is critical for activation of Akt signaling in vivo.

**Figure 5:**
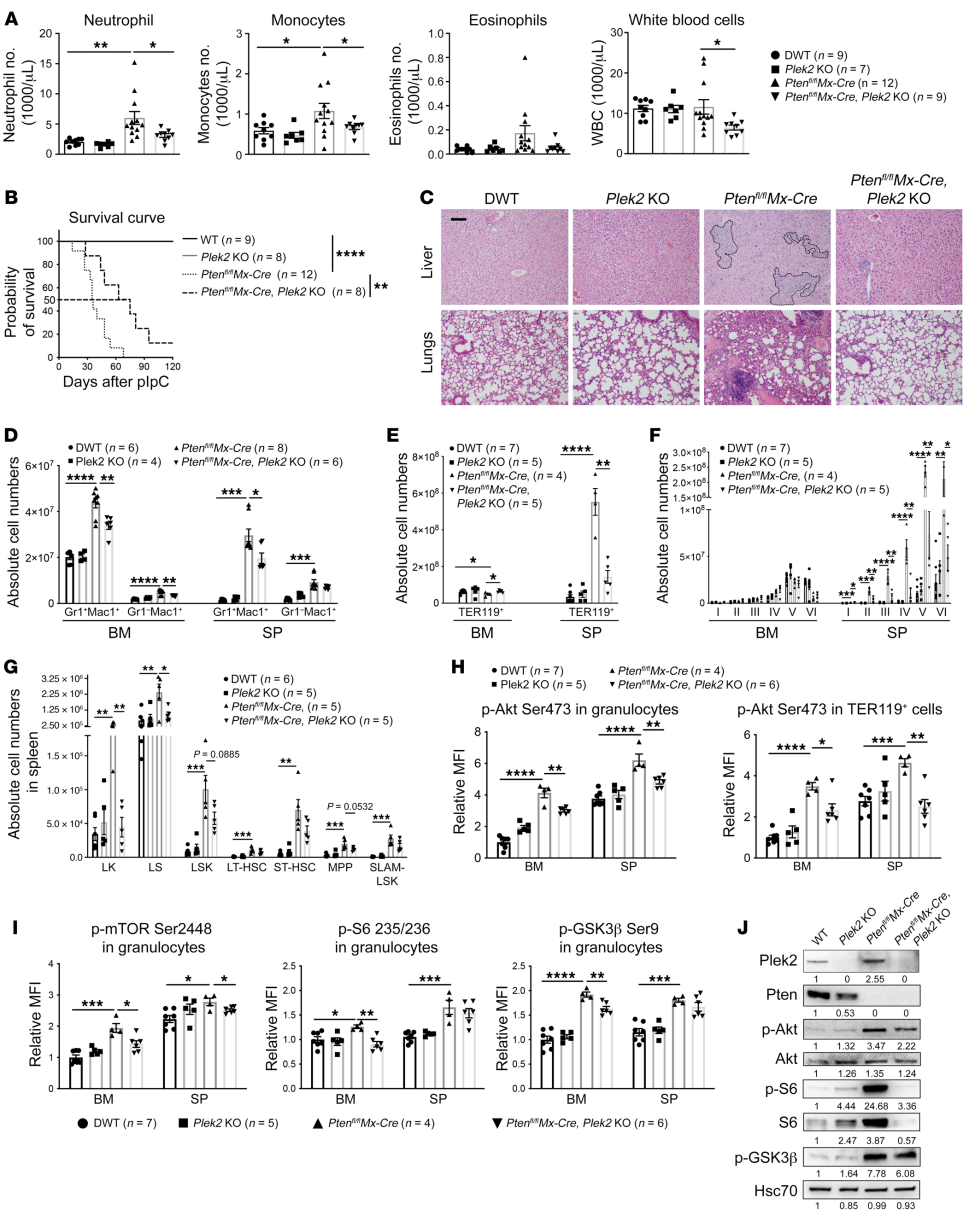
Plek2 is critical for the activation of Akt signaling in vivo. (**A**) Absolute cell counts from indicated mice 25 days after pIpC treatment. DWT, Pten, Plek2 double WT. (**B**) Kaplan-Meier survival assay of indicated mice after pIpC injection. (**C**) Representative H&E staining of liver and lung from indicated mice from **A**. Dashed lines highlight myeloid infiltrates in the liver. Scale bar: 150 μm. (**D**) Absolute cell numbers of the indicated myeloid cells in the BM and spleen (SP) from indicated mice from **A**. (**E** and **F**) Absolute numbers of TER119^+^ erythroid cells in the BM and spleen from indicated mice from **A** are shown in **E**. Further quantitative gating of different maturation stages of erythropoiesis are shown in **F**. Stage I, proerythroblast; stage II, basophilic erythroblast; stage III, polychromatic erythroblast; stage IV, orthochromatic erythroblast; stage V, reticulocytes; stage VI, RBCs. (**G**) Absolute cell numbers of indicated HSPCs from spleens of the indicated mice from **A**. LK, lineage^–^, c-Kit^+^; LS, lineage^–^, Sca1^+^; LSK, lineage^–^, c-Kit^+^, and Sca1^+^; LT-HSC, long-term hematopoietic stem cells; ST-HSC, short-term hematopoietic stem cells; MPP, multipotent progenitors; SLAM-LSK, SLAM^+^ LSK cells. (**H**) Phospho-flow cytometry analyses of p-Akt at serine 473 in the BM and spleen granulocytes and erythroid cells from indicated mice from **A**. (**I**) Phospho-flow cytometry analyses of indicated phosphoproteins in the BM and spleen granulocytes from indicated mice from **A**. (**J**) Western blot assays of indicated proteins from the total BM cells of indicated mice from **A**. Quantification of the proteins is presented below the bands. Error bars represent SEM of the mean. Comparisons between 2 groups were evaluated with 2-tailed *t* test, and comparisons among multiple groups were evaluated with 1-way ANOVA. *P < 0.05; **P < 0.01; ***P < 0.001; ****P < 0.0001.

PTEN is a major negative regulator of PI3K through dephosphorylation of PI3K-generated PI(3,4,5)P_3_ and PI(3,4)P_2_ for inhibiting the PI3K/Akt pathway. PTEN loss of function is common in human cancers through mutations, posttranslational modification, or protein downregulation (32, 33). Loss of Pten in the hematopoietic tissue in mice induces activation of the PI3K/Akt pathway leading to myeloproliferation and lethality approximately 50 days after induced Pten deletion (34, 35). To study the role of Plek2 in the activation of Akt signaling in vivo, we crossed *Pten^fl/fl^Mx-Cre* mice, in which inducible *Pten* deletion primarily in the hematopoietic cells can be achieved through polyinosinic: polycytidylic acid (polyIC) injection, with Plek2-KO mice and obtained *Pten^fl/fl^Mx-Cre*; *Plek2*-KO (DKO Mx-Cre) mice. We then injected polyIC into these mice 7 times when they were 1 month old to induce *Pten* deletion (34). As previously reported, *Pten^fl/fl^Mx-Cre* mice showed myeloproliferative phenotypes, including significantly increased circulating granulocytes and monocytes 25 days after polyIC injection (Figure 5A). The WBC counts were not increased due to the significantly decreased lymphocytes in *Pten^fl/fl^Mx-Cre* mice. These mice also exhibited anemia (Supplemental Figure 6A). Most of the mice died 50 to 60 days after polyIC injection (Figure 5B). As expected, loss of Plek2 significantly reduced the peripheral blood cell count in myeloid lineages and extended the survival of *Pten^fl/fl^Mx-Cre* mice (Figure 5, A and B). Plek2 deficiency also significantly ameliorated BM myeloid proliferation (Supplemental Figure 6B) and myeloid infiltration in multiple organ systems in *Pten^fl/fl^Mx-Cre* mice (Figure 5C and Supplemental Figure 6C). Flow cytometric analyses of the Gr1^+^Mac1^+^ granulocytes and Gr1^–^Mac1^+^ monocytes in the BM and spleen showed marked increase of these cells in *Pten^fl/fl^Mx-Cre* mice that was significantly ameliorated with loss of Plek2 (Figure 5D). The myeloid hyperproliferation was associated with a suppression of BM erythropoiesis and compensatory stress erythropoiesis in the spleen in *Pten^fl/fl^Mx-Cre* mice, which was also significantly reverted with Plek2 deficiency (Figure 5, E and F, and Supplemental Figure 6D). Consistent with previous reports (34, 35), the myeloproliferative phenotype in *Pten^fl/fl^Mx-Cre* mice was initiated from the hematopoietic stem and progenitor cell (HSPC) level. This phenotype was also largely rescued by Plek2 deletion (Figure 5G). Importantly, phospho–flow cytometry analysis revealed that loss of Plek2 largely reverted the increased p-Akt (serine 473) in the BM and spleen granulocytic and erythroid cells induced by *Pten* deletion (Figure 5H). The upregulated downstream signaling of the Akt pathway was also suppressed (Figure 5I and Supplemental Figure 6E). This was further confirmed by a Western blot assay (Figure 5J). These data are consistent with Plek2’s function in all the myeloid lineages and reveal a critical role of Plek2 in the enhancement of the Akt-signaling pathway in vivo that is important for tumorigenesis.

### Plek2 inhibitors are effective in reducing myeloproliferation in vivo.

With the critical roles of Plek2 in the activation of Akt signaling and efficacies of Plek2 inhibitors in vitro, we tested Plek2 inhibitors in vivo in a series of mouse models. We first used an EPO injection model since EPO induces a significant upregulation of Plek2 (12) and it takes less time to show the effect of EPO and the compounds in vivo. Our recently published work using this model demonstrated that repeated injection of EPO rapidly induced erythrocytosis, splenomegaly, and moderate vascular occlusions in WT mice in 3 weeks (12). These phenotypes were significantly ameliorated when the same EPO injections were performed in Plek2-KO mice (12). Before testing NUP-17d in this model, we first determined a no-observed-adverse-effect-level (NOAEL) of the compound to be approximately 25 mg/kg with appropriate absorption, distribution, metabolism, and excretion (ADME) (Supplemental Figure 7A). We treated the mice with NUP-17d after the last EPO injection every day for 3 days. The mice were then sacrificed to determine their complete blood count (CBC), spleen size, and vascular occlusion. Indeed, the compound significantly reduced EPO-induced leukocytosis, reticulocytosis (Supplemental Figure 7, B and C), extramedullary erythropoiesis (Supplemental Figure 7D), and vascular occlusions in the lungs (Supplemental Figure 7E).

**Figure 6:**
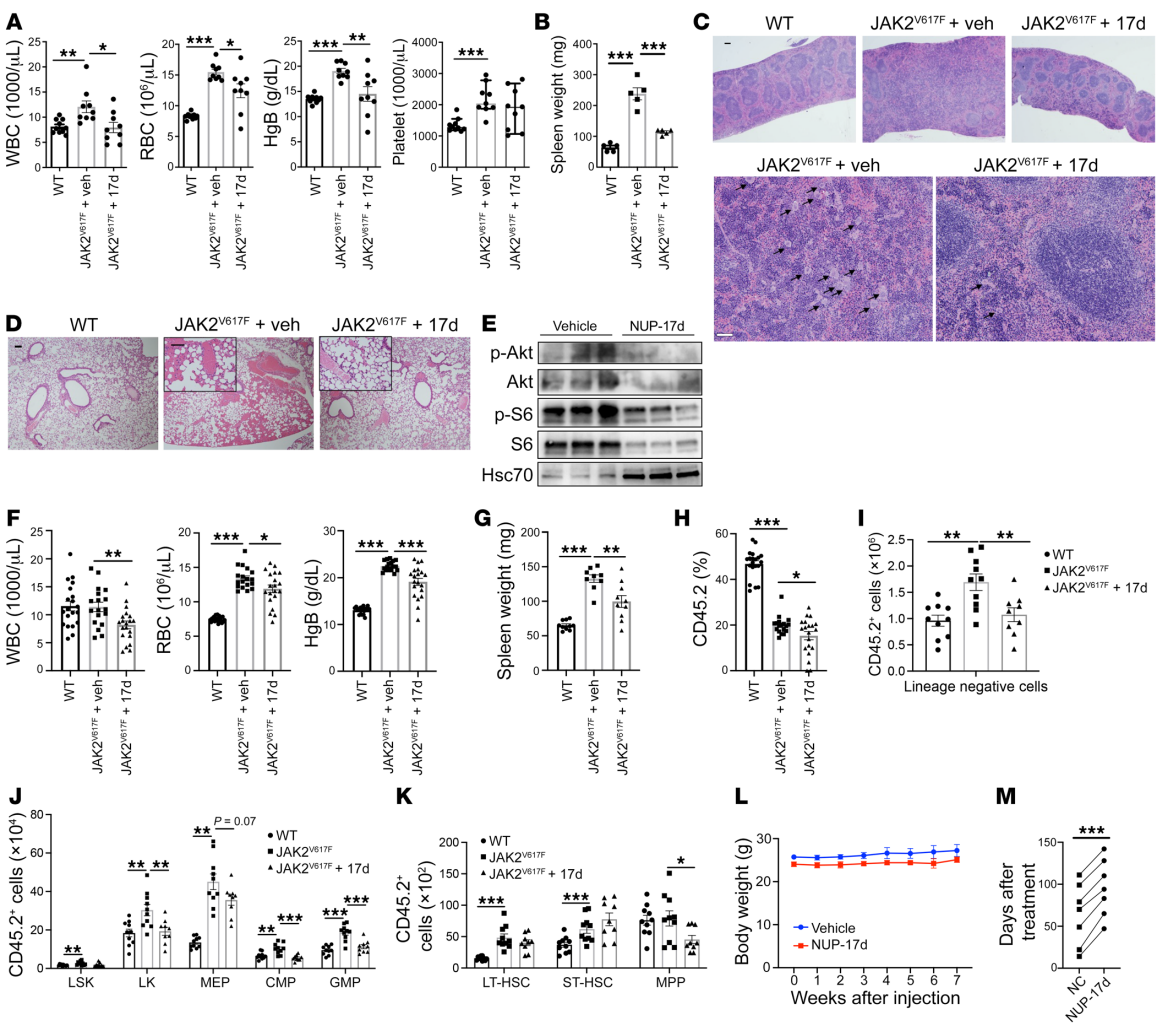
Plek2 inhibitors are effective in reducing myeloproliferation in vivo. (**A**) Total BM cells from 2-month-old JAK2^V617F^-knockin mice (CD45.2^+^) were transplanted into lethally irradiated recipient mice (CD45.1^+^). One month after transplant, recipient mice were treated with 25 mg/kg NUP-17d or vehicle control once every 2 days for 1 month. CBCs were performed after treatment. WT littermate control mice were used for comparison. WT, *n* = 10; *n* = 9 in the other 2 groups. (**B**) Spleen weight in mice from **A**. *n* = 5 in each group. (**C**) Representative H&E staining of spleens from indicated mice after treatment in **A**. Bottom figures are magnified parts of the figures above. Arrows point to megakaryocytes. Scale bars: 100 μm. (**D**) Representative H&E staining of lungs from indicated mice after treatment in **A**. Inserted figures are magnified parts. Scale bars: 100 μm. (**E**) Western blot analyses of indicated proteins from TER119^+^ BM cells purified from mice in **A**. (**F**) Same as **A** except 2-month-old JAK2^V617F^-knockin mice or WT littermates (CD45.2^+^) together with equal amounts (50:50) of WT BM cells (CD45.1^+^) were transplanted. CBCs were performed after treatment. WT (untreated control mice), *n* = 20; JAK2^V617F^+veh, *n* = 18; JAK2^V617F^+17d, *n* = 20. Data were combined from 2 independent experiments. (**G**) Spleen weight in mice from **F**. JAK2^V617F^+Veh, *n* = 9; *n* = 10 in the other 2 groups. (**H**) CD45.2^+^ ratio in peripheral blood from indicated mice from **F**. (**I**–**K**) Absolute CD45.2^+^ cell numbers of indicated HSPCs from BM from indicated mice in **F**. MEP, megakaryocyte-erythrocyte progenitor; CMP, common myeloid progenitor; GMP, granulocyte-monocyte progenitor; WT, *n* = 10; JAK2^V617F^+veh, *n* = 10; JAK2^V617F^+17d, *n* = 9. (**L**) Three-month-old JAK2^V617F^-knockin mice were treated with 25 mg/kg NUP-17d once every week. Same-sex JAK2^V617F^-knockin littermates were treated with vehicle as the control. Body weight was monitored. *n* = 7 in each group. (**M**) Survival data of indicated mice from **I**. Linked 2 points refer to same-sex JAK2^V617F^-knockin littermates. Error bars represent SEM of the mean. Comparisons between 2 groups were evaluated with 2-tailed *t* test, and comparisons among multiple groups were evaluated with 1-way ANOVA. *P < 0.05; **P < 0.01; ***P < 0.001.

With the same dosage, we next applied NUP-17d to a JAK2^V617F^ model. In this model, we transplanted total BM cells from 2-month-old JAK2^V617F^-knockin mice into lethally irradiated recipient mice to ensure consistency in MPN development. All the transplanted mice developed MPN phenotypes 1 month after transplantation (Supplemental Figure 7F). The recipient mice were then treated with NUP-17d or vehicle control for 1 month. As expected, chronic treatment of NUP-17d largely normalized WBC count and significantly reduced erythrocytosis (Figure 6A). Spleen size, extramedullary erythropoiesis, and megakaryocytic hyperplasia were markedly reduced (Figure 6, B and C). Thrombosis in the lungs was also dramatically ameliorated (Figure 6D). Consistent with its role in reducing Plek2 overexpression–mediated activation of Akt signaling, NUP-17d dramatically reduced the levels of p-Akt and S6 in the BM cells from JAK2^V617F^ transplanted mice (Figure 6E). With the same strategy, we also treated recipient mice that were transplanted with equal amounts of BM cells from JAK2^V617F^-knockin and WT mice. NUP-17d not only reduced peripheral cell count and spleen weight as expected (Figure 6, F and G), but it also led to a significant decrease in the JAK2^V617F^ allele burden in the peripheral blood (Figure 6H). We also analyzed whether NUP-17d could also reduce JAK2^V617F^ allele burden in the BM HSPCs. To our surprise, NUP-17d did not reduce the percentage of CD45.2-positive cells (JAK2^V617F^) in different HSPC populations in the BM (Supplemental Figure 7G). However, when we analyzed the absolute count of the CD45.2-positive cells, we found that NUP-17d significantly reduced the cell numbers of most of the HSPCs (Figure 6, I–K). Furthermore, to determine the toxicity profile of the long-term exposure of NUP-17d, we treated 3-month-old JAK2^V617F^-knockin mice with 25 mg/kg NUP-17d once every week for 2 months. In addition to the amelioration of MPN phenotypes (not shown), the long-term treatment did not induce detectable weight loss, but significantly extended the survival of the mice when compared with littermates of the same sex (Figure 6, L and M). With the same strategies, we next treated *Pten^fl/fl^Mx-Cre* mice with NUP-17d and achieved significant reduction in peripheral leukocyte counts (Supplemental Figure 7H) and amelioration of myeloid infiltration in the lungs (Supplemental Figure 7I).

### Plek2 inhibitors synergize with Akt inhibitors and are effective in human MPN cells.

**Figure 7:**
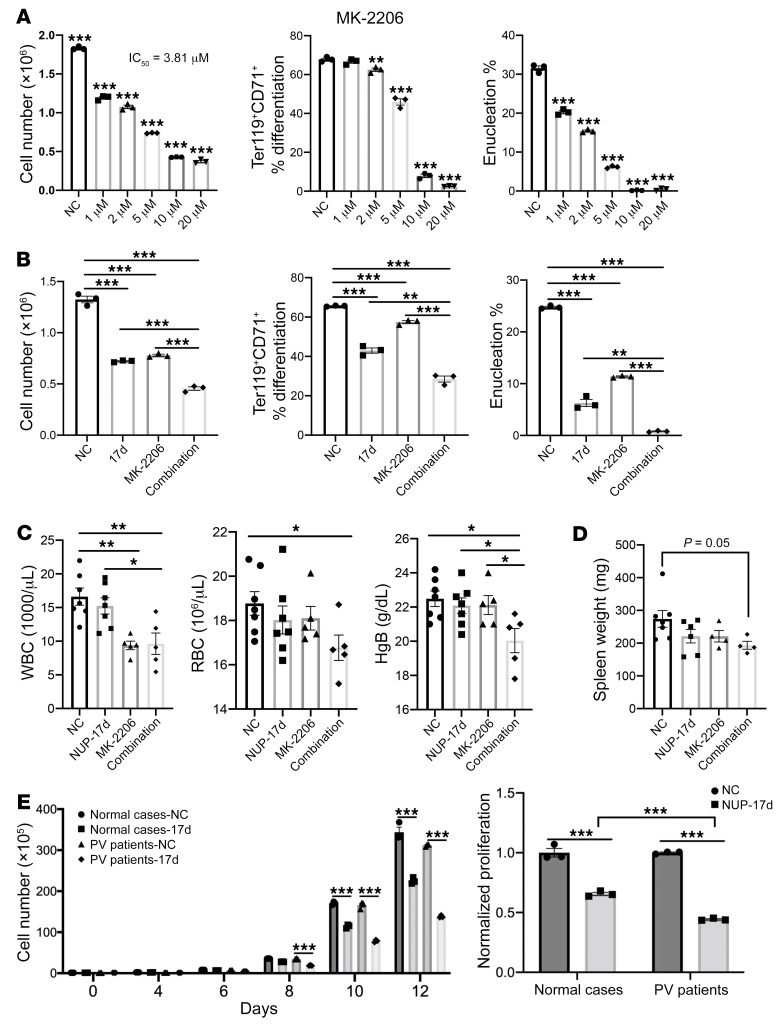
Synergistic effects of combining Plek2 and Akt inhibitors. (**A**) BM lineage–negative cells were cultured in EPO containing medium with different amounts of MK-2206 added at the beginning of culture. Cell proliferation was analyzed at 48 hours in culture. IC_50_ was calculated. Cell differentiation and enucleation were analyzed using flow cytometry. (**B**) BM lineage–negative cells were cultured with NUP-17d (5 M) and MK-2206 (2 μM) added at the beginning of culture. Cell proliferation, differentiation, and enucleation were analyzed at 48 hours in culture. Calculated CI was 0.73, 0.54, and 0.49 for cell proliferation, differentiation, and enucleation, respectively. (**C**) Total BM cells from 2-month-old JAK2^V617F^-knockin mice (CD45.2^+^) were transplanted into lethally irradiated recipient mice (CD45.1^+^). One month after transplant, recipient mice were treated with 25 mg/kg NUP-17d, 25 mg/kg MK-2206, or a combination of NUP-17d and MK-2206 with 25 mg/kg each for 2 weeks. Peripheral blood indices were analyzed. NC (vehicle control), *n* = 7; NUP-17d, *n* = 7; MK-2206, *n* = 5; combination, *n* = 5. (**D**) Spleen weight of mice in **C**. NC (vehicle control), *n* = 7; NUP-17d, *n* = 6; MK-2206, *n* = 4; combination, *n* = 4. (**E**) CD34-positive cells purified from healthy individuals or patients with PV were cultured in EPO medium and treated with DMSO (NC) or 10 μM NUP-17d every 2 days. Cells were counted at indicated time points. Data are representative of 3 independent experiments from 3 healthy individuals and 3 patients with PV. Data directly comparing the sensitivity to NUP-17d in these groups on day 12 are presented on the right. Error bars represent SEM of the mean. Comparison between 2 groups were evaluated with 2-tailed *t* test, and comparisons among multiple groups were evaluated with 1-way ANOVA. *P < 0.05; **P < 0.01; ***P < 0.001.

These in vivo efficacy studies indicate the therapeutic potentials of Plek2 inhibitors in MPNs. The JAK inhibitor ruxolitinib has been approved for clinical use in MPNs, while many Akt inhibitors are under clinical investigation. Patients with both types of therapies suffer considerable side effects (36, 37). In this respect, the negligible phenotypes of the Plek2-KO mice (12) and minimal toxicity profiles of Plek2 inhibitors suggest Plek2 targeted therapy as a promising alternative strategy. Plek2 inhibitors could also be used for combination therapies, with Akt inhibitors given the critical roles of Plek2 in Akt signaling. To test this, we first determined the IC_50_ levels of MK-2206, an allosteric Akt inhibitor, in our erythroid culture system. MK-2206 showed slightly enhanced inhibitory effects in proliferation and enucleation compared with NUP-17d. There was a significant inhibition of cell differentiation for MK-2206, indicating potential toxicity (Figure 7A). When we combined NUP-17d with MK-2206 in the same in vitro assay, the combination treatment was synergistic and more effective than the inhibitory effect of each individual compound (Figure 7B). We next treated the mice transplanted with JAK2^V617F^ BM cells with combination therapy and reduced doses of NUP-17d. Compared with the single compound treatment, combination treatment led to a significant reduction in blood cell count and spleen weight (Figure 7, C and D) and amelioration of thrombosis and pathology in the BM and spleen (Supplemental Figure 8A). Furthermore, the combination therapy also mildly, but statistically significantly, reduced the allele frequency of JAK2^V617F^ in an in vitro assay (Supplemental Figure 8B).

To determine the effect of Plek2 inhibitors in cells from MPN patients, we purified CD34-positive hematopoietic progenitor cells from 3 normal individuals and 3 patients with PV (a subtype of MPNs; patients’ clinical information is presented in Supplemental Table 3) and cultured them separately in medium promoting erythroid differentiation and proliferation. Indeed, cells from PV patients were more sensitive to the inhibitory effects of NUP-17d (Figure 7E).

### Generation of Plek2 inhibitors with enhanced specificity.

NUP-17d has 2 chiral centers and is determined to be a mixture of 4 stereoisomers (2 diastereoisomers, each consisting of 2 enantiomers) (data not shown). This could cause nonspecific binding to other proteins, especially in Plek2-deficient cells. We separated these stereoisomers by high-performance liquid chromatography (HPLC) and supercritical fluid chromatography (SFC). We used x-ray crystallographic analysis and determined the absolute configuration of each stereoisomer. We then performed a series of medicinal chemistry studies and obtained newer compounds NUP-17d-8A and NUP-17d-52A, which lack the chiral centers. Both compounds showed specific binding to Plek2 with high affinity (Supplemental Figure 9, A and B). CETSA and differential scanning fluorimetry melting assay further confirmed the binding (Supplemental Figure 9, C and D). Both compounds, especially NUP-17d-52A, also showed strong inhibition of erythroid proliferation in vitro (Supplemental Figure 9, E and F). To determine the specificity of the optimized inhibitors, we used lineage-negative BM cells from Plek2-KO mice and tested NUP-17d-52A in the erythroid proliferation system. Plek2-deficient erythroblasts showed significantly reduced proliferation capacity compared with the WT counterparts. When treated with NUP-17d-52A, the Plek2-deficient cells were also significantly less sensitive compared with the WT cells (Supplemental Figure 9G). These results demonstrate that the newer compounds are more specific to Plek2 and provide a platform for continued optimization and drug development.

## Discussion

Our study reveals that Plek2 functions to form a membrane-signaling complex to recruit the effector proteins in the PI3K/Akt pathway and enhance Akt signaling. This complex also recruits heat shock protein Hsp72 to stabilize Akt. Since Plek2-KO mice do not exhibit significant phenotypes, the role of Plek2 in proproliferation is best manifested in the context of Plek2 overexpression, which is observed in MPNs and many solid tumors. These findings suggest Plek2’s role as a proto-oncoprotein that warrants future studies. The binding of the Plek2 inhibitor to Plek2 disrupts this signaling complex and leads to the destabilization and inactivation of Akt. The negligible phenotypes in Plek2-KO mice also enable Plek2 inhibitors as a class of anticancer agents with possible fewer side effects.

Aberrant activation of Akt signaling in cancers makes Akt an important target in drug development. However, because of the critical roles of Akt in various cellular processes, small-molecule compounds directly targeting Akt often result in severe side effects (38). In this respect, targeting Plek2 could be an attractive alternative approach, since Plek2 is not essential for survival (12). In MPNs, Plek2 is upregulated by the hyperactivated JAK2/STAT5 pathway. The JAK2/STAT5 pathway is commonly activated in hematologic malignancies (39), suggesting a broader application of Plek2 inhibitors in cancers. The upregulation of Plek2 in solid cancers could also be mediated through the JAK2/STAT5 pathway, but other upstream regulators likely control the expression of Plek2 as well.

Like Plek1, Plek2 contains 2 PH domains, which is rare among PH domain–harboring proteins. The strong membrane association of Plek2 facilitates its recruitment of additional PI3K/Akt signaling components and heat shock protein to form a stable membrane signaling complex. Plek2 itself is likely stabilized in this complex, since disruption of the complex by Plek2 inhibitors reduces its protein levels. Previous studies also demonstrated that PI3K regulates Plek2 in T cell cytoskeletal reorganization (19). The role of Plek2 in regulating actin cytoskeleton is also evident in both fibroblast cell lines and primary erythroid cells (11). Since PI3K is well known as playing an important role in inducing actin filament remodeling through Akt and its downstream effectors (40), these functions of PI3K could also be mediated through Plek2 as a D3 phosphoinositide membrane-anchoring protein to coordinate these signaling events.

Since many proteins contain the PH domain, we chose to use the DEP domain on Plek2 to screen its binding small molecules. Through binding of the DEP domain, NUP-17d disrupts Plek2’s functions as a regulatory protein to recruit PI3K/Akt signaling effector proteins and Hsp72 to stabilize the complex. The critical residues on Plek2 DEP domain for the binding to Plek2 inhibitors are quite specific in DEP domain–containing proteins, indicating that Plek2 inhibitors may have fewer off-target effects. This is further supported by the fact that Plek2 inhibitors showed no effects on the basal levels of lamellipodia in Cos-7 cells or nonproliferative erythroid cells. However, the possibility that Plek2 inhibitors bind to other targets cannot be excluded, especially under a hyperproliferative condition in the absence of Plek2. This is especially concerning since the first-generation Plek2 inhibitor NUP-17d is a mixture of 4 stereoisomers. Indeed, the newer generation of more pure compounds (such as NUP-17d-52A) showed increased specificity. Future structure-activity relationship–guided (SAR-guided) medicinal chemistry studies will be required to develop more potent and specific Plek2 inhibitors.

In addition to the capability of reducing peripheral blood cell count, NUP-17d also reduced JAK2^V617F^ allele burden in the peripheral blood manifested by the decreased percentage of CD45.2-positive cells in a cotransplantation model (Figure 6, F–K). Surprisingly, we did not observe the same reduction of CD45.2 percentages in BM HSPCs of the treated mice. This could be related to the short period of treatment. Mature blood cells in the peripheral blood could also be more sensitive to Plek2 inhibitors, since Plek2 levels gradually increase during blood cell differentiation (12). In addition, it is notable that JAK2^V617F^ decreases the CD45.2 percentage, which has been shown before due to the impaired hematopoietic stem cell function (41). The same reduction in HSPC fitness was also previously observed in the JAK2^V617F^-knockin model used in our current study (42). Therefore, the increased total BM cellularity in the cotransplantation model is also contributed to markedly by the expanded CD45.1-positive WT cells. The fact that NUP-17d reduced CD45.2-positive JAK2^V617F^ cell numbers but failed to reduce its percentage demonstrates that NUP-17d also decreases the number of the expanded CD45.1 WT cells. It is well established that JAK2^V617F^ mutant cells influence the nonmalignant BM and stromal cells in MPNs (43). How mutant cells drive the proliferation of their WT counterparts remains an intriguing topic for further investigation. Our data indicate that Plek2 inhibitors are also effective in reverting these influences on the WT cells.

In the current study, we also found that the potency of the Plek2 inhibitor is comparable to that of a well-studied Akt inhibitor while the toxicity profile of Plek2 inhibitor is favorable. This is consistent with the dispensable roles when it is not overexpressed. One of the current major bottlenecks in the development of Akt inhibitors is the side effects. These could be caused by the inhibitors’ effects on different Akt isoforms and the complex mechanisms of Akt regulation and crosstalk to other pathways. Therefore, Plek2 inhibitors provide an alternative approach to targeting the Akt pathway as single agents or in combination with Akt inhibitors with reduced doses. Future clinical studies using Plek2 inhibitors in various Akt-activated cancers would be informative.

## Methods

### Mice.

WT mice (C57BL/6) for the drug screen and EPO injection assays were purchased from The Jackson Laboratory. CD45.1^+^ WT mice for BM transplantation were purchased from Charles River Laboratories. Jak2^V617F^ floxed (*Jak2^VF/+^*) hematopoietic-specific knockin mice (*Jak2^VF/+^Vav-Cre*) (originally reported in ref. 42), *Plek2*-KO, and *Jak2^VF/+^Vav-Cre*, *Plek2*-KO mice were previously reported (12). Pten floxed (*Pten^fl/fl^*) mice were purchased from The Jackson Laboratory. These mice were crossed with *Mx-Cre* mice (The Jackson Laboratory) to generate hematopoietic-specific Pten-KO mice. These mice were further crossed with Plek2-KO mice to generate *Pten^fl/fl^Mx-Cre, Plek2*-KO mice. To test the CBCs, peripheral blood (75 μL from each mouse) was collected from retroorbital veins in EDTA-treated minicollection tubes (Greiner Bio-One GmbH). The Hemavet 950 complete blood counter (Drew Scientific) was used to determine CBCs. For pathologic analyses, the leg bones, spleen, and lungs were collected from euthanized mice and treated with formalin solution (MilliporeSigma) before H&E staining.

### Homology model building of DEP domain of Plek2 and in silico screening.

Using the primary amino acid sequence of the DEP domain of Plek2 as the query, relevant template structures were first identified by performing a homology search in the Protein Data Bank (PDB) database with BLAST and PSI-BLAST. The search did not yield a single template with more than 30% sequence similarity to the DEP domain query sequence. Hence, a multiple-template technique utilizing various templates was used to build the model. The templates were assigned to different regions of the query sequence of the DEP domain of Plek2. Hence, each part of the DEP domain was built with the appropriate template structure. The Prime 3.1 module of the Schrödinger suite (44) was used for building the homology model. Prime 3.1 is a well-validated protein structure–prediction suite of programs that integrates comparative modeling and fold recognition into a single interface. The comparative modeling path incorporates complete protein structure prediction, including template identification, alignment, and model building. Furthermore, Prime allows for refinement of the side-chain prediction, loop prediction, and minimization. The alignment steps were then used to align the templates with the query. Unfortunately, the alignment was not optimal and had several gaps. Secondary structure prediction (SSP) tools were used to obtain better alignment. After building the model of Plek2, it was validated using MolProbity (45) software, version 4.2. The MolProbity analysis revealed that the Plek2 model built in Prime and minimized using the OPLS-2005 force had less than 2% clash score, less than 3 % poor rotamers, less than 0.80% Ramachandran outliers, and 96% favorable rotamers, and there were no residues with bad bonds and angles. The site identification (SITE-ID) module (46) implemented in Sybyl X1.3 was used to identify potential small-molecule, ligand-binding sites in Plek2.

### In silico filtering of a commercially available small-molecule database.

The ZINC database, which contains approximately 50 million compounds, was used for vHTS (47). All compounds were subjected to a panel of PAINs and SMARTS filters (48, 49) to eliminate molecules with non–drug-like characteristics that interfere with functionality, including reactive functional groups and known toxophores. This filtering generated a list of approximately 100,000 drug-like commercially available compounds that were subjected to further screening.

### In silico screening workflow.

The Glide docking engine (50) implemented in the Schrödinger suite was utilized to carry out the 100,000 compound screening. The Prime-generated and Desmond-simulated (50) Plek2 model was prepared in the Optimized Potentials for Liquid Simulations (OPLS 2005) force field. The Glide docking engine is built on a grid-based algorithm. After preparing the Plek2 model in OPLS-2005, the grid-generating submodule was used to create a 12 × 12 × 12 Å grid box centered on the small-molecule, ligand-binding site identified earlier using SITE-ID. The ligand preparation tool available in the Schrödinger suite was used to protonate/deprotonate the 100,000-compound set at pH =7.4 ±1 and to obtain the best initial geometry of the compounds. The ligand van der Waals radii were scaled to 0.80 Å with partial atomic charges of less than 0.15 esu. The Glide screening workflow has a 3-tier docking process consisting of vHTS, standard precision (SP), and finally, extra precision (XP) docking protocols. The 3-tier docking engine generated 59 hits with Glide docking scores less than –6.0. The docking score is a function of the binding energy of the compounds (44). All 59 structures were crossvalidated by docking using a different docking engine in order to obtain consensus scores for the hit molecules. The cross validation used the Surflex docking engine, which is a tool built on a fragment-based algorithm implemented in Tripos software and therefore orthogonal to the grid-based algorithm used by Glide (46). Based on the consensus scores from both the docking experiments, 32 in silico hits were selected. From this set, 28 compounds were purchased for screening in biological assays based on commercial availability, pricing, and synthetic tractability.

### BM transplantation.

BM transplantation was performed as previously reported (51, 52). For the Jak2^V617F^ transplantation model, total BM cells from 2-month-old *Jak2^VF/+^Vav-Cre* mice (CD45.2^+^) were harvested and transplanted into lethally irradiated (10 Gy) WT recipient mice (CD45.1^+^). For competitive BM transplantation, total BM cells from 2-month-old *Jak2^VF/+^Vav-Cre* mice or WT littermates (CD45.2^+^) were equally mixed (50:50 ratio) with WT BM cells (CD45.1^+^) and transplanted into lethally irradiated recipient mice (CD45.1^+^). One month after transplant, the recipient mice were as indicated in the text.

### Cell culture and in vitro erythroid differentiation.

Cell culture and in vitro erythroid differentiation were performed as described previously (23, 24, 53).

For the in vitro compound treatment assay, compounds or DMSO control was added with indicated concentrations at the beginning of culture and was used to treat the cells for 48 hours. After treatment, cell number was calculated on a Z1 Particle Counter (Beckman Coulter). Differentiation and enucleation were analyzed by flow cytometry. All the compound information is in Supplemental Table 1, except that MK-2206 was purchased from Selleck. The combination index (CI) was calculated as follows: CI = (D)1/(Dχ)1 + (D)2/(Dχ)2. (Dχ)1 and (Dχ)2 represent concentrations of each compound alone to exert χ% effect. (D)1 and (D)2 are concentrations of compounds in combination to elicit the same effect. CI < 1, = 1, and > 1 indicate synergism, additivity, and antagonism, respectively.

### Flow cytometric assays.

Single-cell suspensions of cultured cells were prepared by resuspending the cells in PBS with 0.5% BSA (Santa Cruz Biotechnology Inc.) and 2 mM EDTA (Gibco; Thermo Fisher Scientific). Cells were immunostained with the following antibodies purchased from eBioscience: CD71 (catalog 11-0711), Ter119 (catalog 17-5921), CD44 (catalog 12-0441), CD45.2 (catalog 48-0454), B220 (catalog 17-0452), Gr-1 (catalog 48-5931), CD41 (catalog 11-0411), CD11b (catalog 17-0112), CD117 (catalog 17-1171-82), and CD3e (catalog 48-032). Cells were also immunostained with the following antibodies from BioLegend: Sca1 (catalog 108108), CD135 (catalog 135310), CD117 (catalog 105814 and 105820), CD150 (catalog 115910), and CD16/CD32 (catalog 101323). Hochest33342 (Invitrogen) was used to stain nucleated cells. Propidium iodide was added to exclude dead cells from analysis. All staining was performed for 30 minutes at room temperature.

For the phospho–flow cytometric assay, single-cell suspensions from the BM or spleen were fixed in 4% paraformaldehyde dissolved in PBS at room temperature for 15 minutes followed by PBS washing. The cell pellets were recovered after centrifugation and resuspended in PBS. The prechilled True-Phos Perm Buffer (BioLegend) was added to permeabilize cells. Cells were then incubated at –20°C overnight to ensure proper permeabilization. Cells were then washed in PBS followed by centrifugation at 1,000*g* at room temperature for 5 minutes. Samples were blocked with HBSS/4% FBS at 4°C for 1 hours. Sufficient cells were aliquoted and stained at room temperature. Samples were then directly stained in the dark for 30 minutes with fluorescence-labeled antibody cocktails (1:200 dilution), including PE-Pten (BD Biosciences), PE–p-S6 (Ser235, Ser236) (eBioscience), PE–p-mTOR (Ser2448) (eBioscience), PE–p-AKT (Thr308) (Cell Signaling Technology), and APC–p-Akt (Ser473) (Cell Signaling Technology). For detection of phosphorylation of GSK3b and Foxo3a, samples were first stained with primary antibodies (1:200 dilution, Cell Signaling Technology). After washing, samples were incubated with APC-labeled secondary antibody (1:200 dilution, Thermo Fisher Scientific) for an additional 30 minutes in the dark. All samples were washed by PBS prior to data acquiring. All flow cytometric analysis was performed using a BD LSRFortessa X-20 flow analyzer, and results were further analyzed with FlowJo software, version 10.3.0 (TreeStar Inc.).

### Western blotting analysis.

Whole cell lysates were extracted with RIPA buffer (Pierce, Thermo Scientific) containing protease inhibitor and PhosphStop cocktail (Roche). The total protein concentration was measured with a BCA kit according to the manufacturer’s instructions (Thermo Scientific). For Western blotting, the following antibodies were used: anti-HSC70 (catalog sc-7298) (Santa Cruz Biotechnology Inc.); anti-Pten (catalog 9559), anti-total AKT (catalog 4691), anti-phospho AKT (catalog 4060), anti-total S6 (catalog 2217), anti-phospho S6 (catalog 4858), anti-phospho GSK3b(catalog 5558), anti-mTOR (catalog 2972), anti-GFP (catalog 2956), anti-GST (catalog 2622), anti-Flag (catalog 8146), and anti-HA (catalog 3724) (Cell Signaling Technology); anti-Plek2 (catalog 11685-1-AP) and anti-PDK2 (catalog 15647-1-AP) (Proteintech); anti-Hsp72 (catalog PA5-34772) (Invitrogen); and HRP linked anti-GST antibody (catalog MA4-004-HRP) (Thermo Fisher Scientific). The Western blot results were further analyzed using Image Lab software (Bio-Rad). 

### Expression and purification of GST-tagged proteins and GST pull-down assay.

pGEX-6p3-Plek2 (GST-Plek2), pGEX-6p1-Plek2 DEP domain (GST-DEP; amino acids 128 to 224), pGEX-6p1-Plek2 DEP triple mutations (GST-DEP 3M, amino acids 128 to 224, K156N/S162A/R194G), and pGEX-6p1-Akt (GST-Akt) were constructed according to the manufacturer’s instructions (Invitrogen). The constructs were transfected into BL21-competent cells (New England Biolabs) to express the fusion proteins induced by 0.2 mg/mL IPTG (MilliporeSigma). The GST-tagged proteins were purified with magnetic glutathione particles according to the manufacturer’s instructions (Promega). Purified His-AKT protein was purchased from Abcam. GST pull-down assays between GST-Plek2 or GST-DEP and His-AKT were performed according to the manufacturer’s instructions (Promega). For pull-down assays with biotin-tagged compound, streptavidin magnetic beads were coincubated with biotin-NUP-17d and GST-Plek2 or GST-Plek2 DEP truncation mutant according to the manufacturer’s instructions (Pierce; Thermo Scientific).

### Transfection plasmids and co-IP assays.

PCMV-tag2B-Flag-Plek2 (Flag-Plek2), PCMV-tag2B-Flag-Plek2 triple mutations (Plek2 3M, Plek2 full length, K156N/S162A/R194G), and MICD4-HA-Plek2 (HA-Plek2) were constructed according to the manufacturer’s instructions (Invitrogen). GFP-Hsp72 (catalog 19483), HA-Akt (catalog 78778), HA-Akt (aa 1–149) (catalog 73410), HA-Akt (aa 120-433) (catalog 73411), HA-GST-Akt (catalog 48805), HA-Ubi-K48 (catalog 17605), HA-Ubi-K63 (catalog 17606), and HA-Ubi (WT) (catalog 17608) were purchased from Addgene. BirA-Plek2 was constructed by GenScript Biotech Corp. For transfection, 2 μg total plasmids were transfected into HEK293T cells in 6-well plates using TransIT-LT1 transfection reagent (Mirus) according to the manufacturer’s protocol. After 24 hours or 48 hours, co-IP assays were performed using anti-FLAG, anti-HA magnetic beads, or streptavidin magnetic beads according to the manufacturer’s instructions (Pierce; Thermo Scientific) or anti-GST according to the manufacturer’s instructions (Promega). The precipitated samples were further analyzed by Western blotting as described above. For the BirA-Plek2 assay (streptavidin-biotin IP/pulldown), the specific band (around 70 kDa) was cut off after Western blot and further analyzed using mass spectrometry. The mass spectrometry results are in Supplemental Table 2.

### Generation of retrovirus and infection of lineage-negative cells.

Generation of retrovirus and infection of lineage-negative cells were performed as previously reported (12). In brief, 6 μg MICD4-Plek2 shRNA or control vector and 3 μg packaging construct pCL-Eco were cotransfected into HEK293T cells using TransIT-LT1 transfection reagent (Mirus). Viral supernatants were collected 48 hours after transfection. For the infection, lineage-negative cells were incubated with viral supernatants in the presence of 8 μg/ml polybrene (MilliporeSigma) and centrifuged at 400*g* for 90 minutes at 37°C. The viral supernatants were gently removed after spin infection, and the cells were incubated with fresh EPO medium and further cultured for 24 to 48 hours in vitro.

### Lipid-binding assays.

The binding assay between GST-Plek2 and the lipid array strip was performed according to the manufacturer’s instruction (Echelon Biosciences). The binding assays using lipid-coated beads with or without compound treatment were performed according to the manufacturer’s instruction (Echelon Biosciences).

### Mass spectrometry assays.

Mass spectrometry was used to measure PI(3,4,5)P_3_ in TER119-positive erythroblasts (1 × 10^6^) as previously described (54) using a QTRAP 4000 (AB Sciex) mass spectrometer and employing the lipid extraction and derivatization method described for cultured cells. PIP_2_ regio-isomers (PI[3,4]P_2_ and PI[4,5]P_2_) of the primary lipid species in TER119-positive cells, stearoyl/arachidonoyl (C18:0 C20:4), were quantitated in parallel samples (1 × 10^6^) employing previously described methods (26). Data are presented as mean ± SD from 3 separate experiments. Data are shown as pmol/10^6^ cells, calculated by normalizing the targeted lipid integrated response area to that of a known amount of relevant internal standard (pg lipid). Data were then converted to pmol based on the molecular weight of the relevant lipid species (pg/pmole). The quantitative proteomics of TMT mass spectrometry assay was performed as previously reported (52).

### CETSA.

CETSA was performed as previously reported (55). In brief, Flag-Plek2 was transfected into HEK293T cells using TransIT-LT1 transfection reagent according to the manufacturer’s instructions (Mirus). Cells were collected 48 hours after transfection, and cell numbers were counted; 5 × 10^5^ cells were resuspended in 100 μL PBS after PBS wash and then treated with 10 M indicated compound or DMSO control (2%) for 1 hour on ice. Cells were then washed 3 times with cold PBS and resuspended in 50 μL PBS. After incubation at indicated temperatures for 3 minutes, cells were treated with 3 cycles of liquid nitrogen, freezing and thawing on ice. The whole cell lysate was harvested after centrifugation at 1,600*g* and 4°C for 15 minutes and further analyzed by Western blot as described above.

### ITC assay.

Samples for ITC experiments were prepared in PBS with 1% DMSO. All experiments were performed on a MicroCal ITC200 instrument (Malvern Panalytical) in the Keck Biophysics Facility at Northwestern University. Before each experiment, the reference cell and syringe were filled with deionized filtered water, then washed extensively with the reaction buffer. The sample solutions were degassed for 10 minutes; then 40 μL of the drug solution was loaded into the titrating syringe and 280 L of the protein solution was placed in the ITC cell. After the instrument was equilibrated at 25°C and 300*g* syringe rotational speed, a first injection of 0.1 μL was performed followed by a series of 1.5 μL injections. The first injection was discarded from the analysis of the integrated data to avoid artifacts related to diffusion through the injection port during the equilibration period. To measure residual heats, a separate control experiment was performed for each of the compounds tested. This consisted of 10 injections of the compound solution into buffer with the experimental settings described above. ITC data were processed with MicroCal Origin 7.0 software package as described (56). Individual injection heats (qi) — obtained by integrating the corresponding injection peaks — were normalized for ligand concentration and corrected for dilution heats. Nonlinear regression fit to a single set of sites model (57) provided the stoichiometry of binding N, equilibrium association constant Ka, and enthalpy change ΔH for each experiment.

### RNA-Seq and GSEA.

HSPCs purified from the BM of WT mice were cultured in EPO-containing medium and treated with Plek2 inhibitor as described above. After treatment, total RNAs were extracted using TRIzol reagent following the manufacturer’s recommendations (Invitrogen). RNA quality and quantity were determined using an Agilent Bioanalyzer. RNA-Seq libraries were generated using an Illumina TruSeq Stranded mRNA Kit per the manufacturer’s instructions. Libraries were quantified using an Agilent Bioanalyzer. Sequencing was performed using an Illumina HiSEQ4000 with Illumina-provided reagents and protocols. RNA-Seq data were deposited in the NCBI’s Gene Expression Omnibus database (GEO GSE176397). GSEA was performed using RNA-Seq gene expression profiling data and GSEA software (Broad Institute).

### MPN patient sample.

CD34-positive cells from the BM aspirate were obtained using antibody-mediated positive selection followed by purification. CD34^+^ cells were then cultured in StemSpan SFEM Medium (Stem Cell Technology) supplemented with 10% FBS (Stem Cell Technology), 10 ng/mL IL-3 (Stem Cell Technology), 50 ng/mL SCF (Stem Cell Technology), and 2 U/mL recombinant human EPO (Amgen Inc.) from day 0 to day 6. After day 6, cells were cultured in StemSpan SFEM Medium supplemented with 30% FBS and 2 U/mL EPO.

### In vivo drug treatment assays.

The EPO induction mouse model used in a short-term drug treatment assay was previously reported (12). Briefly, 5,000 IU/kg EPO or PBS was intraperitoneally injected once every 2 days into WT mice for 2 weeks. Plek2 inhibitors were then injected intraperitoneally into these mice once every day for 3 days. For long-term drug treatment assays, Jak2^V617F^ transplantation mice were used. For Plek2 inhibitor treatment of *Pten^fl/fl^Mx-Cre* mice, polyIC was intraperitoneally injected once every 2 days for 25 days to induce hematopoietic-specific Pten KO, which was followed by Plek2 inhibitor treatment of 25 mg/kg detailed in the text. The formula for Plek2 inhibitor solution is 10% DMSO, 2% ethanol, 2% Tween 80 (MilliporeSigma), and 86% of 0.5% Captisol solution (CyDex Pharmaceuticals).

For the survival assay after drug treatment, *Jak2^VF/+^Vav-Cre* mice were injected with indicated compound or compounds, followed by the treatment strategies detailed in the text. Mice were monitored daily and sacrificed when moribund. For the survival assay of *Pten^fl/fl^Mx-Cre* mice and *Pten^fl/fl^Mx-Cre*, *Plek2*-KO mice, animals were monitored daily and sacrificed when moribund. Statistical analysis was performed with Prism (GraphPad Software) via the Mantel-Cox or Gehan-Breslow-Wilcoxon test.

See supplemental material for full, uncut gels.

### Statistics.

All statistics analyses were performed with Prism 8 (GraphPad Software), and all data are represented as mean ± SD. Comparisons between 2 groups were evaluated with 2-tailed *t* test, and comparisons among multiple groups were evaluated with 1-way ANOVA. Significance was defined as a *P* value of less than 0.05.

### Study approval.

MPN patient samples were obtained from leftover diagnostic specimens at the Department of Pathology, Northwestern University. The study protocol was approved by the institutional review board at Northwestern University. All animal experiments were conducted in accordance with the Guide for the Care and Use of Laboratory Animals (National Academies Press, 2011) and approved by the Institutional Animal Care and Use Committee at Northwestern University.

## Author contributions

XH, YM, RKM, HB, ADJ, BZ, MAP, PW, AAG, JAP, MS, KEA, LS, JY, and PJ performed experiments and interpreted data. ADJ, GES, PW, JIP, and PJ performed medicinal chemistry, characterized the compounds, and analyzed data. XH, YM, CSA, JY, and PJ analyzed data. XH and PJ designed experiments, interpreted data, and wrote the manuscript.

## Supplementary Material

Supplemental data

Supplemental table 1

Supplemental table 2

Supplemental table 3

## Version 1 01/31/2023

In-Press Preview

## Version 2 03/15/2023

Electronic publication
