# Supplementary material for: Targeting pleckstrin-2/Akt signaling reduces proliferation in myeloproliferative neoplasm models
Source: J Clin Invest. 2023 Mar 15;133(6):e159638. doi: 10.1172/JCI159638 (PMC10014099; doi:10.1172/JCI159638)

Supplemental Figures

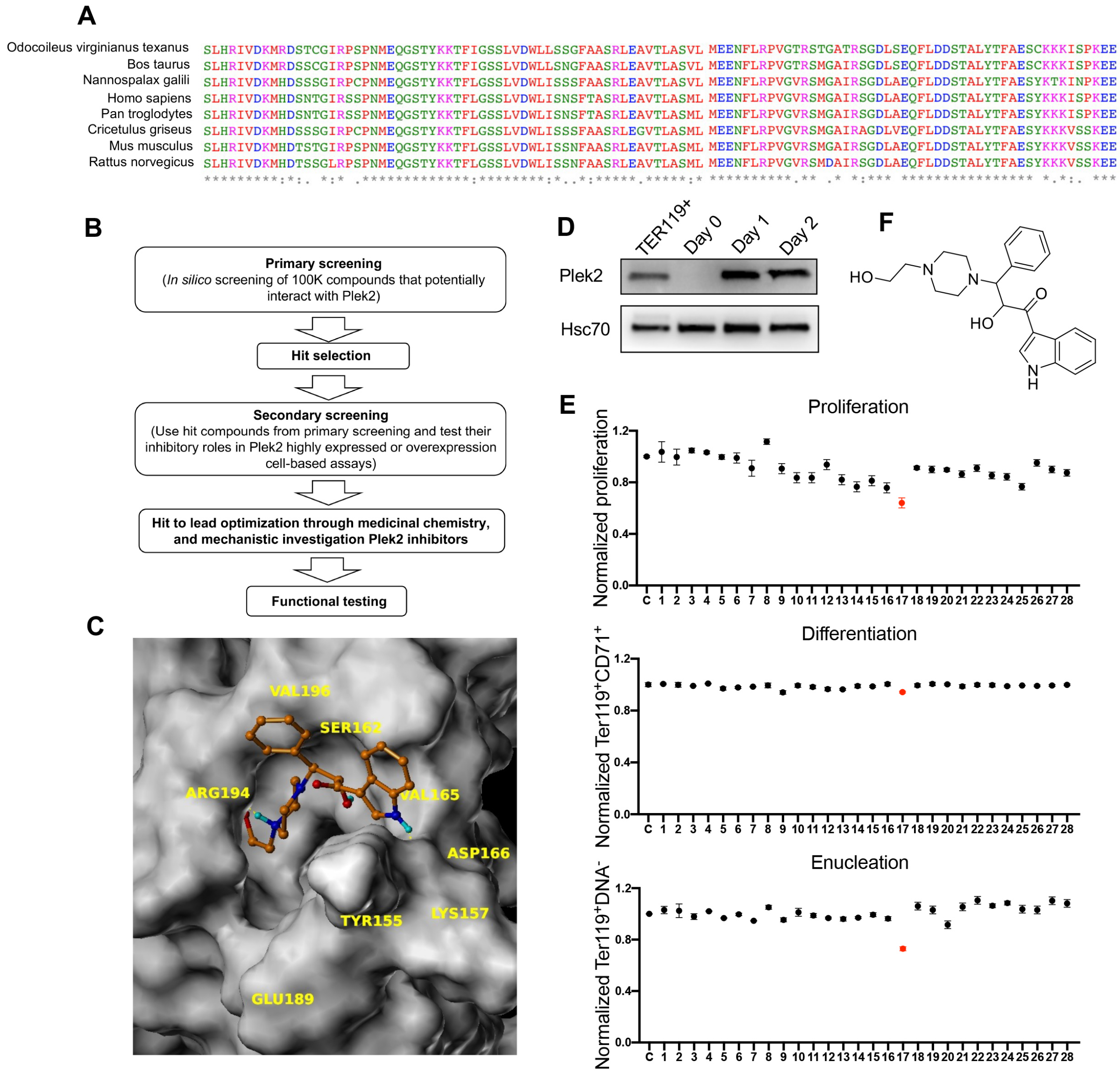

Supplemental Figure 1. Development of Plek2 small molecule inhibitors. A, Phylogenetic sequence alignment (CLUSTAL omega) shows highly conserved Plek2 DEP domain (amino acids 128-236). An asterisk (*) denote fully conserved residue; a colon (:) denotes conservation between groups of similar properties; and a period (.) denotes conservation between groups of weakly similar properties. **B,** Screening strategy of Plek2 small molecule inhibitors. **C,** Docking model of NUP-17 and Plek2 DEP domain. Critical amino acids for the interaction on the DEP domain are highlighted in yellow. **D,** Western blotting assay of indicated proteins from wild type bone marrow lineage negative cells cultured in EPO-containing medium for the indicated time. TER119+ erythroid cells freshly isolated from the bone marrow were also tested. **E,** Wild type bone marrow lineage negative cells were cultured with 10 μM of the indicated hit compounds added at the beginning of culture. Cell proliferation, differentiation and enucleation were analyzed at 48 h in culture. **F,** Structure of NUP-17.

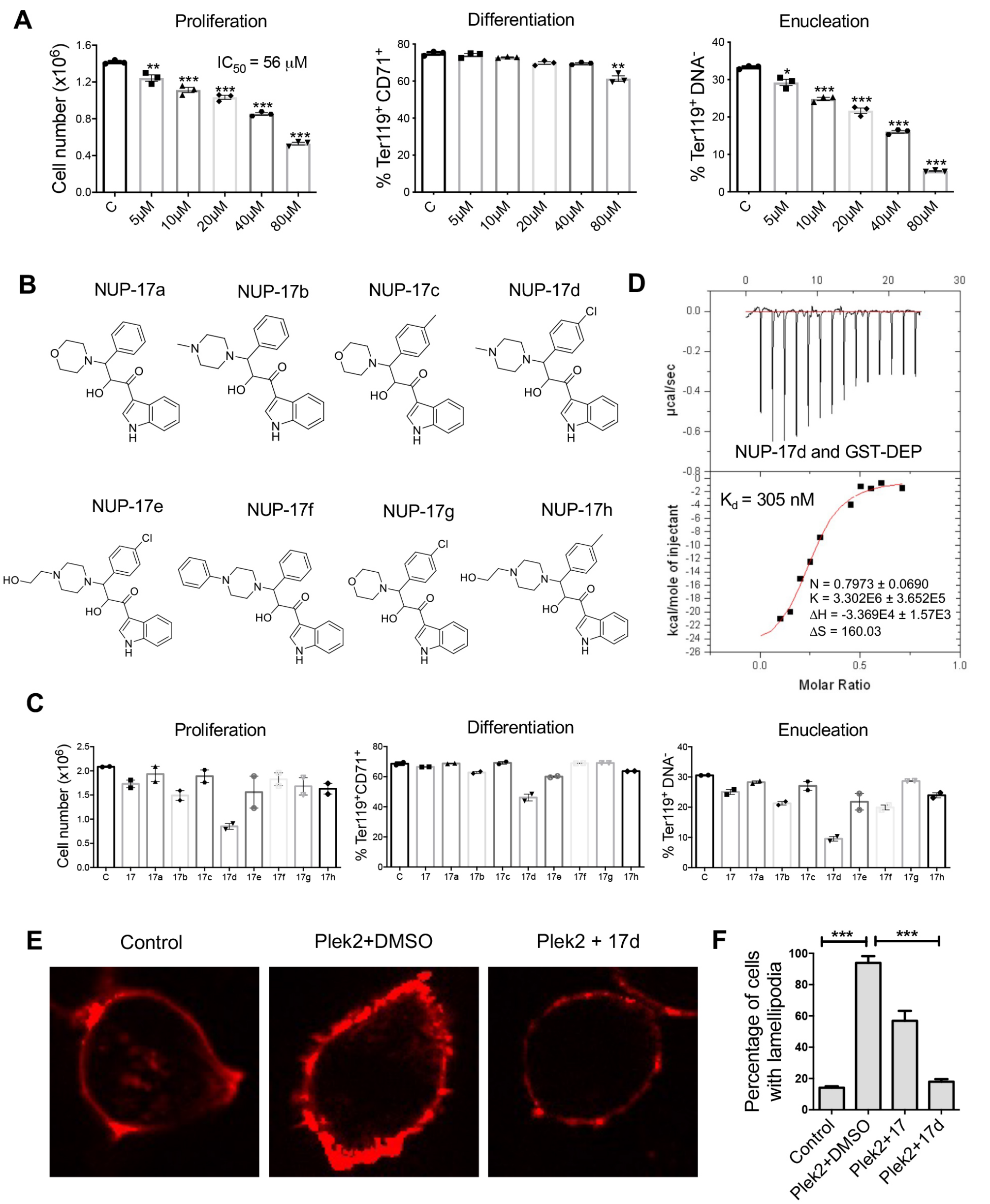

Supplemental Figure 2. Identification of initial lead compounds of Plek2 inhibitors. A, Wild type bone marrow lineage negative cells were cultured with different amount of NUP-17 added at the beginning of culture. Cell proliferation was analyzed at 48 h in culture. The half maximum inhibitory concentration (IC_{50}) was calculated. Cell differentiation and enucleation were analyzed using flow cytometry. **B,** Structure of NUP-17 analogs. **C,** Wild type bone marrow lineage negative cells were cultured with the indicated NUP-17 analogs (10 μM) added at the beginning of culture. Cell proliferation, differentiation and enucleation were analyzed at 48 h in culture as in A. **D**, Isothermal titration calorimetry (ITC) analyses demonstrate a direct interaction of NUP-17d with Plek2 DEP domain. **E**, Plek2 was overexpressed in Cos-7 cells followed by the treatment of 10 μM NUP-17d for 24 h. Phalloidin 647 was used to stain actin. **F,** Statistic analyses of E. All the error bars represent the SEM of the mean. The comparison between two groups was evaluated with 2 tailed t test and the comparison among multiple groups was evaluated with 1-way ANOVA test. * $p<0.05$, ** $p<0.01$, and *** $p<0.001$.

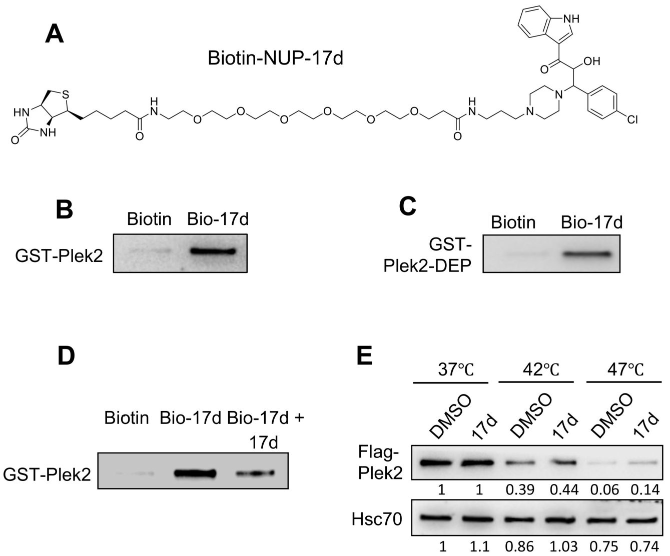

Supplemental Figure 3. NUP-17d directly binds to Plek2. **A**, Chemical structure of biotinylated NUP-17d. **B-C**, Biotin or biotinylated NUP-17d was incubated with GST-Plek2 (B) or GST-Plek2-DEP (C) followed by a pull-down experiment using streptavidin beads. Western blot assays were performed with anti-Plek2 or anti-GST. **D**, Same as B except with or without the presence of NUP-17d (500 μM). **E**, Cellular thermal shift assay to test binding of NUP-17d with Plek2 in the cells. 293T cells transfected with Flag-Plek2 were treated with 10 μM NUP-17d on ice for 1 h followed by incubation at indicated temperature for 3 min. Western blotting assay was then performed for Flag-Plek2. Quantification of the proteins is presented below the bands.

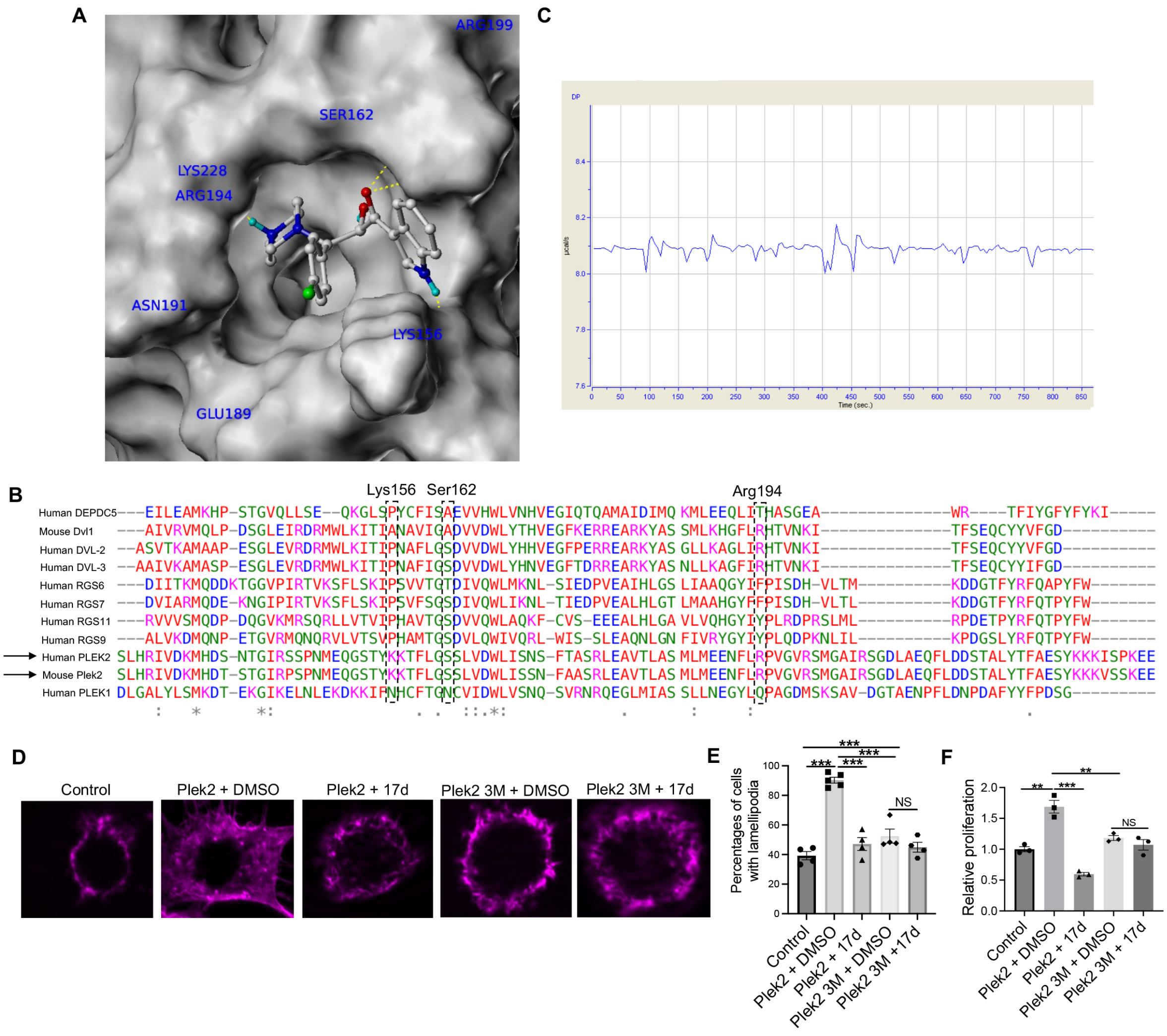

Supplemental Figure 4. Specific residues on Plek2 DEP domain critical for the binding to NUP-17d. A, Docking model of NUP-17d and Plek2 DEP domain. Critical amino acids for the interaction on the DEP domain are indicated with yellow dotted lines. **B**, Sequencing alignment of DEP domains from the indicated proteins. Lys156, ser162, and arg194 are highlighted. **C**, Isothermal titration calorimetry (ITC) analysis demonstrates no interaction of NUP-17d with mutant Plek2 DEP. **D**, Plek2 or Plek2 3M was overexpressed in Cos-7 cells followed by the treatment of 10 µM NUP-17d or DMSO as indicated for 24 h. Deep red/Cy5 was used to track actin. **E**, Statistic analyses of D. The error bars represent the SEM of the mean. The comparison was evaluated using 1-way ANOVA test. ***$p<0.001$. **F**. HEL cells were transduced with Plek2, Plek2 3M, or the control construct, and then treated with NUP-17d for 48 h at the concentration of 10 µM. CCK-8 assays were performed to analyze relative proliferation.

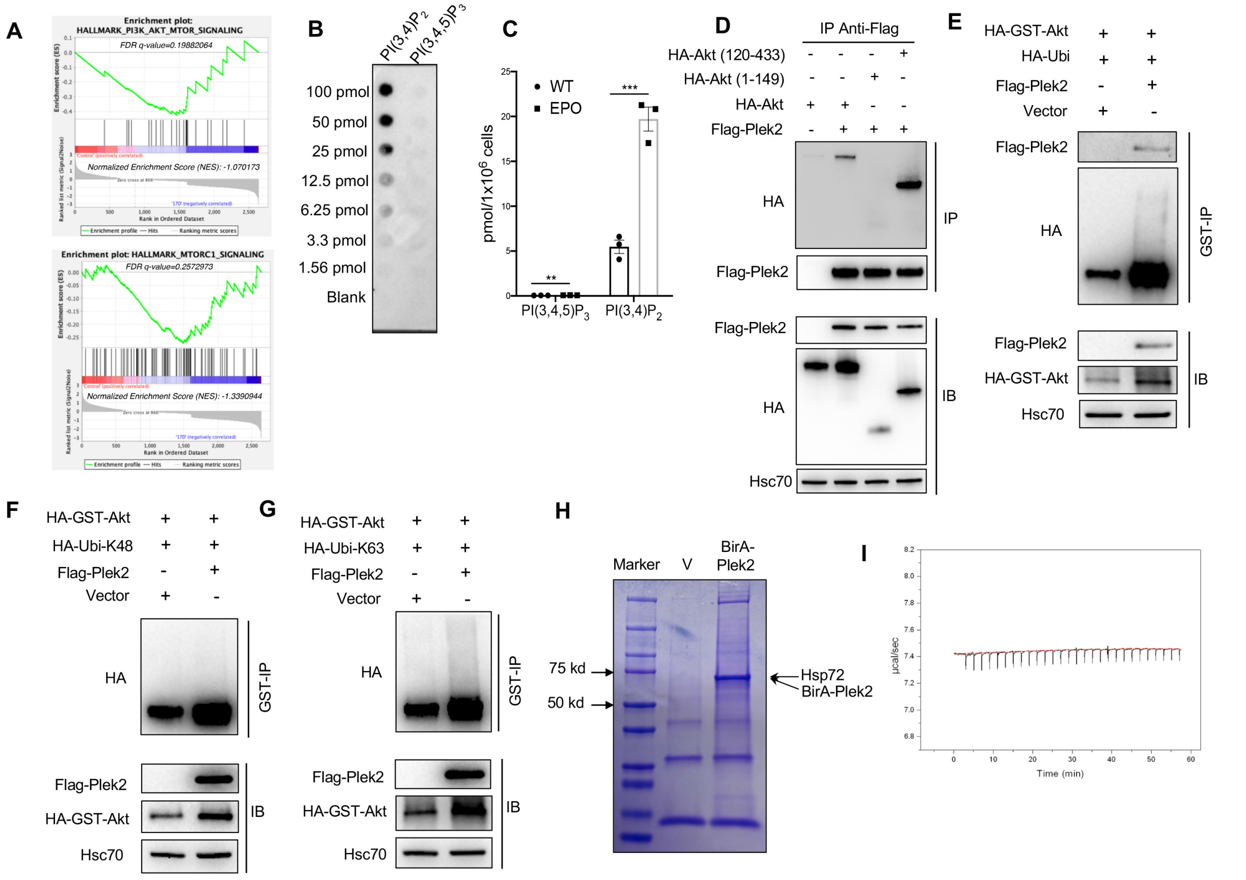

Supplemental Figure 5. Plek2 stabilizes Akt through Hsp72. A, Gene set enrichment analysis (GSEA) of the RNA sequencing analyses in Figure 2A shows PI3K-AKT-MTOR signaling and MTORC1 signaling were downregulated. **B**, GST-Plek2 (1 µg/mL) was incubated with different concentrations of PI(3,4)P2 and PI(3,4,5)P3 followed by a Western blotting assay of GST. **C**, Quantitative analysis of the cellular content of indicated phosphoinositide with or without EPO stimulation of bone marrow TER119+ erythroblasts. **p<0.01; ***p<0.001. **D**, Immunoprecipitation of Flag tag with cell lysate from 293T cells transfected with Flag-Plek2, HA-Akt or HA tagged Akt truncation mutants followed by a Western blotting assay of indicated proteins. IP: immunoprecipitation; IB: immunoblotting. **E-G,** Anti-GST immunoprecipitation with cell lysate from 293T cells transfected with Flag-Plek2, HA-GST-Akt, HA-Ubiquitin (E), HA-Ubiquitin K48 (F), or HA-Ubiquitin K63 (G) followed by Western blotting assays of indicated proteins. All the lysine residues are mutated to arginine except K48 and K63 in F and G, respectively. **H,** Bead-conjugated streptavidin pull down of 293T cells transfected with BirA-Plek2 and incubated with biotin (50 µM), followed by an SDS-PAGE and Coomassie stain. Proteins enriched in BirA-Plek2 transfected cells were identified via mass spectrometry. **I,** Isothermal titration calorimetry (ITC) analysis demonstrates no interaction of NUP-17d with recombinant Akt.

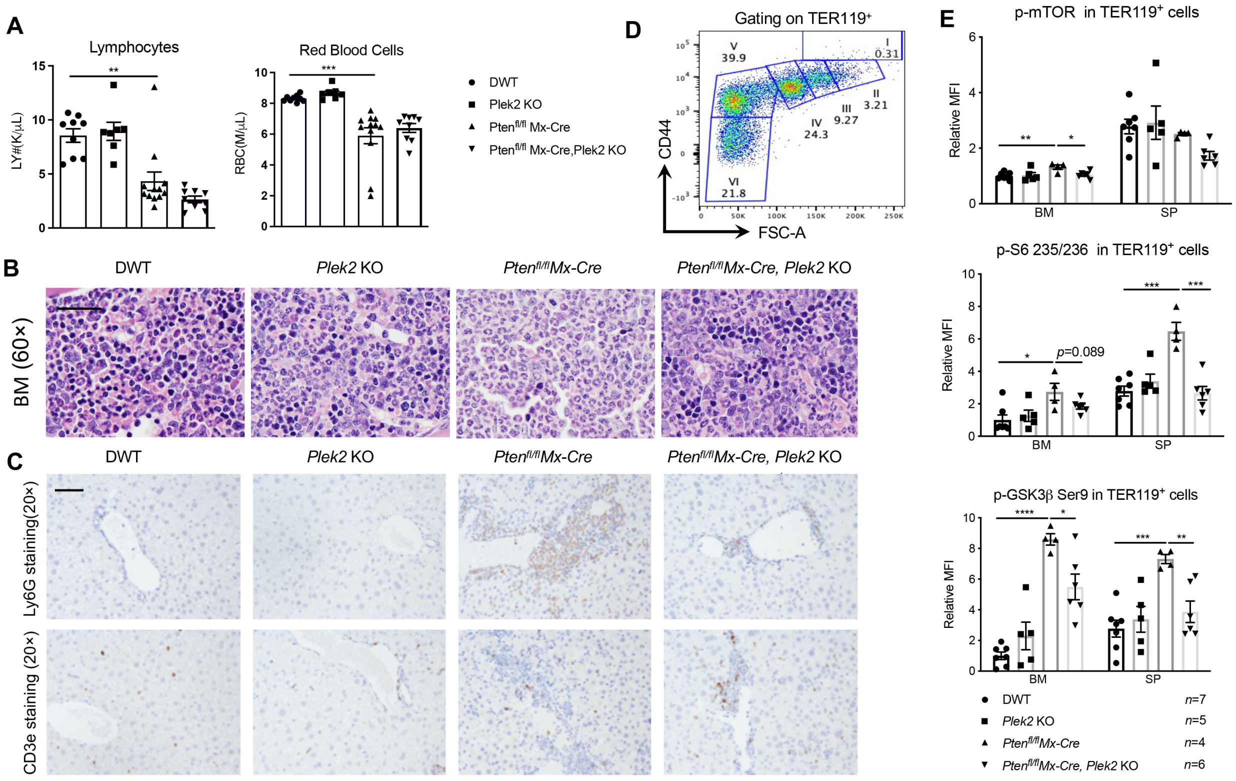

Supplemental Figure 6. Plek2 is critical for the activation of Akt signaling *in vivo*. A, Lymphocyte and red blood cell counts from indicated mice 25 days after pIpC treatment. DWT: PTEN, Plek2 double wild type. **B,** Representative hematoxylin and eosin stain of the bone marrow from indicated mice 25 days after pIpC injection. Cells with condensed dark blue nuclei are erythroblasts, which are markedly reduced and replaced by myeloid cells in the bone marrow of *Pten$^{fl/fl}$Mx-Cre* mice. The Scale bar: 100 μm. **C,** Representative immunohistochemistry stain of Ly6G and CD3e from indicated mice 25 days after pIpC injection. Scale bar: 100 μm. **D,** Gating strategy of different maturation stages of erythropoiesis. **E,** Phospho-flow cytometry analyses of indicated phosphoproteins in the bone marrow and spleen TER119+ cells from indicated mice 25 days after pIpC injection.

All the error bars represent the SEM of the mean. The comparison between two groups was evaluated with 2 tailed t test and the comparison among multiple groups was evaluated with 1-way ANOVA test. * p<0.05, **p<0.01, ***p<0.001 and ****p<0.0001.

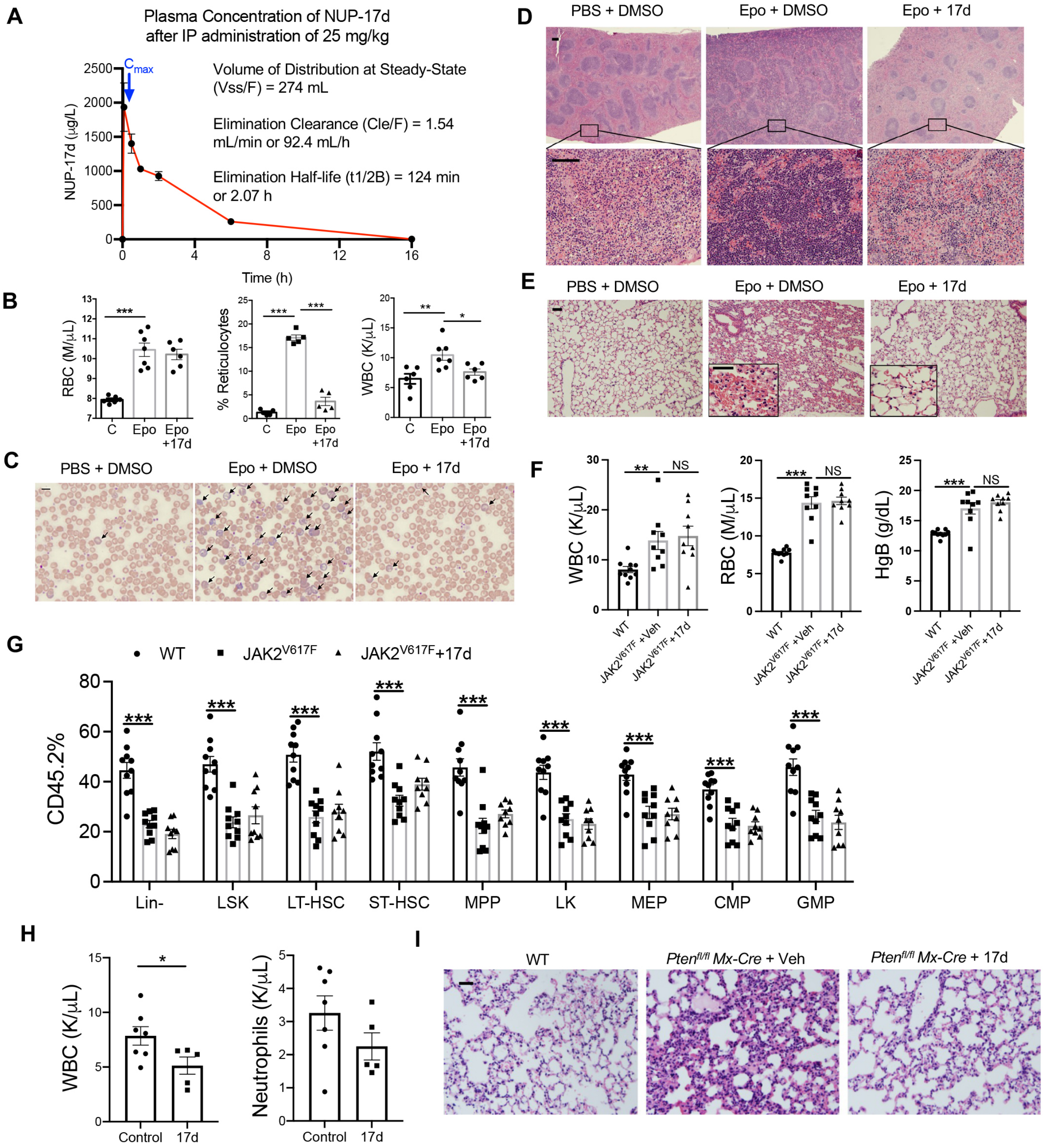

Supplemental Figure 7. Plek2 inhibitors are effective *in vivo*. A, Drug metabolism and pharmacokinetics of NUP-17d after intraperitoneal administration of 25 mg/kg into two-month-old wild type mice. **B,** Two-month-old wild type mice were intraperitoneally injected with 5000 IU/kg erythropoietin once every two days for two weeks. The mice were then injected with 25 mg/kg NUP-17d or vehicle control once every day for three days. Complete blood counts were performed after the treatment. For WBC and RBC counts, n=7 in control (C) and erythropoietin (Epo) injection groups; n=6 in Epo plus NUP-17d group. n=5 in each group for reticulocyte

percentages in peripheral blood. **C,** Representative Giemsa stain of peripheral blood smear from indicated mice in B. Scale bar: 10 μm. **D,** Representative hematoxylin and eosin stain of the spleen from indicated mice in B. Scale bars: 100 μm. **E,** Representative hematoxylin and eosin stain of the lungs from indicated mice in B. Inserted figures were magnified parts. Scale bars: 100 μm. **F**, Total bone marrow cells from two-month-old JAK2^{V617F} knockin mice (CD45.2+) were transplanted into lethally irradiated recipient mice (CD45.1+). Complete blood counts were performed one month after transplantation before the indicated groups to be treated. Wild type littermate control mice were used for comparison. WT: n=10; n=9 in the other two groups. **G**, CD45.2+ ratio in the bone marrow HSPCs from indicated mice in Figure 6F. **H,** Hematopoietic specific Pten deficient mice were treated with vehicle control or 25 mg/kg NUP-17d once every two days for two weeks. Peripheral blood white blood cell and neutrophil counts were analyzed after the treatment. **I**, Representative H&E stains of the lungs from the indicated mice. Scale bar: 100 μm.

All the error bars represent the SEM of the mean. The comparison between two groups was evaluated with 2 tailed t test and the comparison among multiple groups was evaluated with 1-way ANOVA test. * $p<0.05$, ** $p<0.01$, and *** $p<0.001$.

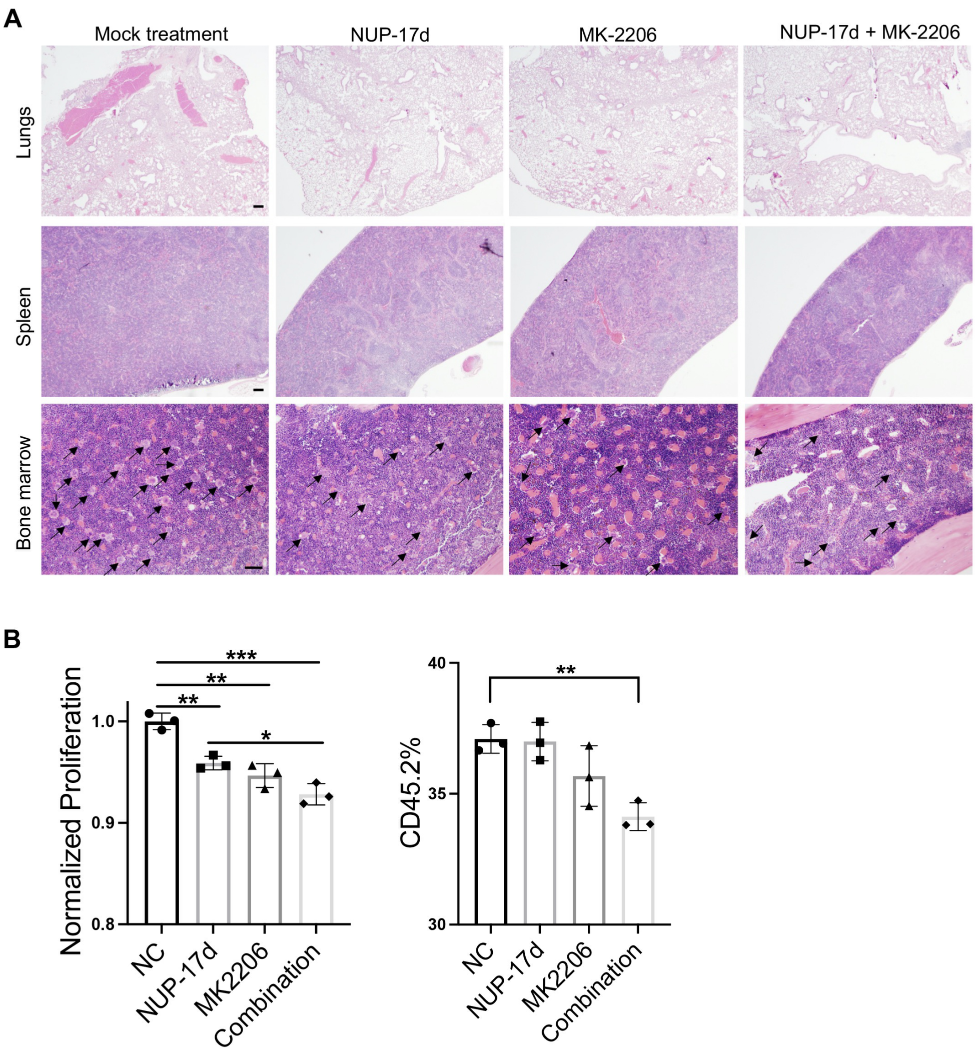

Supplemental Figure 8. Synergistic effects of combing Plek2 and Akt inhibitors. A, Representative hematoxylin and eosin stain of the lungs, spleens and bone marrow from indicated mice after treatment in C. Scale bars: 100 μm. Arrows indicate megakaryocytes. **B**, Equal number of bone marrow c-Kit+ cells from JAK2^{V617F} knockin (CD45.2+) and wild type control (CD45.1+) mice were co-cultured for 24 h followed by the treatment of the indicated compounds for 48 h. Cell number and the percentage of CD45.2+ cells were measured. NUP-17d: 5 μM; MK-2206: 2 μM.

All the error bars represent the SEM of the mean. The comparison between two groups was evaluated with 2 tailed t test and the comparison among multiple groups was evaluated with 1-way ANOVA test. * $p<0.05$, ** $p<0.01$ and *** $p<0.001$.

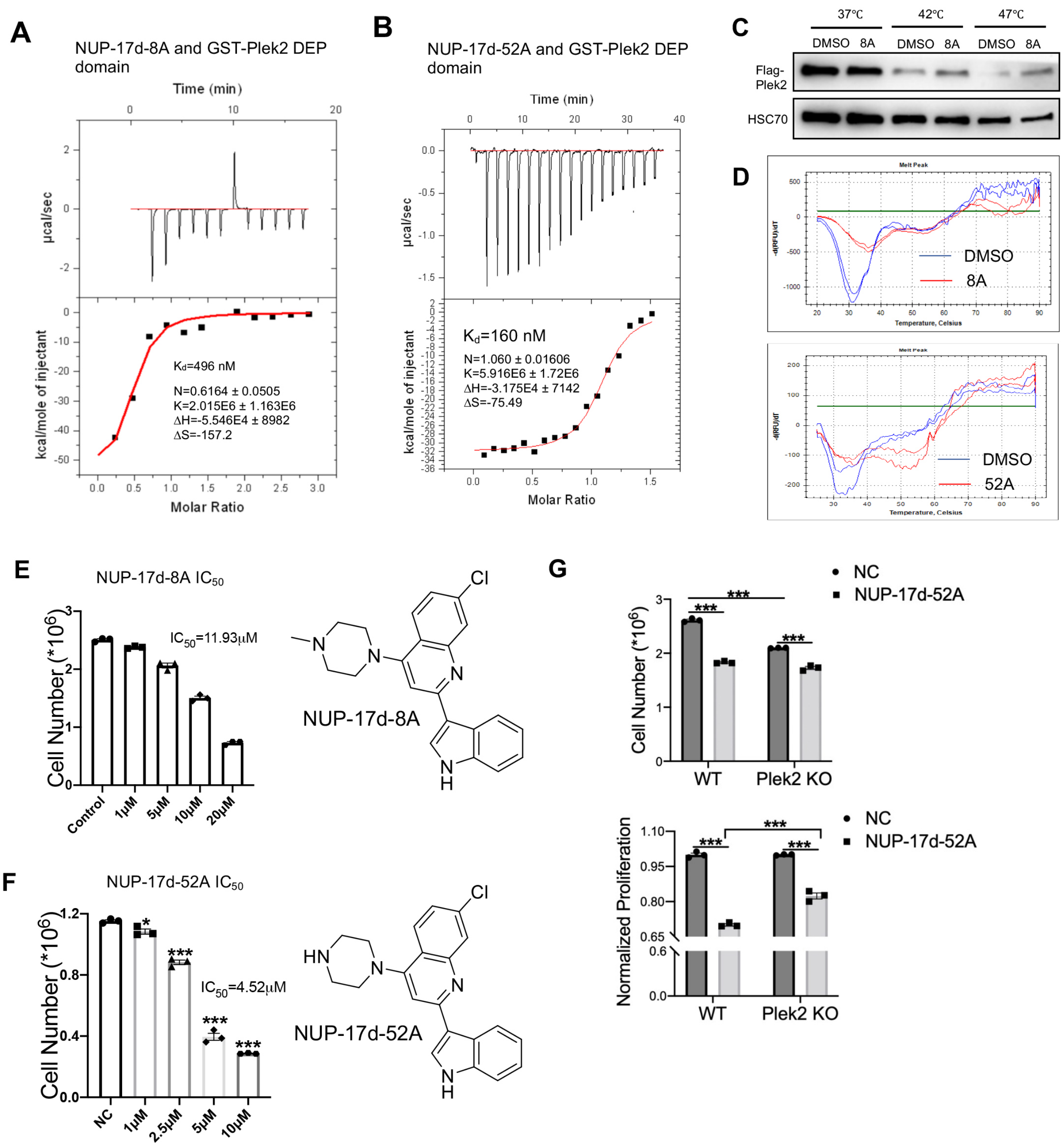

Supplemental Figure 9. Improved Plek2 inhibitors with increased potency and specificity. A, Isothermal titration calorimetry (ITC) analyses demonstrate direct interaction of NUP-17d-8A with DEP domain of Plek2. **B**, Isothermal titration calorimetry (ITC) analyses demonstrate direct interaction of NUP-17d-52A with DEP domain of Plek2. **C**, Cellular thermal shift assay to test binding of NUP-17d-8A with Plek2 in the cells. 293T cells transfected with Flag-Plek2 were treated with 10 µM NUP-17d-8A on ice for 1 h and followed by incubation at indicated temperature for 3 min. Western blotting of Flag-Plek2 was then performed. **D**, Differential scanning fluorimetry melting data demonstrate interaction of NUP-17d-8A (upper) and NUP-17d-

52A (down) with recombinant human Plek2. **E**, Wild type bone marrow lineage negative cells were purified and cultured with different amount of NUP-17d-8A added at the beginning of culture. Cell proliferation was analyzed at 48 h in culture. The half maximum inhibitory concentration (IC_{50}) was calculated. NUP-17d-8A structure is shown on the right. **F**, Same as E except NUP-17d-52A was tested. **G**, Bone marrow lineage negative cells from 2-month-old wild type (WT) or Plek2 knockout (KO) mice were purified and cultured with 3 μM NUP-17d-52A added at the beginning of culture. Cell proliferation was analyzed at 48 h in culture. The upper panel shows the exact cell number. The bottom panel shows the proliferation rate normalized on vehicle treatment group (NC). All the error bars represent the SEM of the mean. *$p < 0.05$, **$p < 0.01$, ***$p < 0.001$. Two-tailed unpaired Student's t test was used to generate the p values.

Full unedited gel for Figure 1I

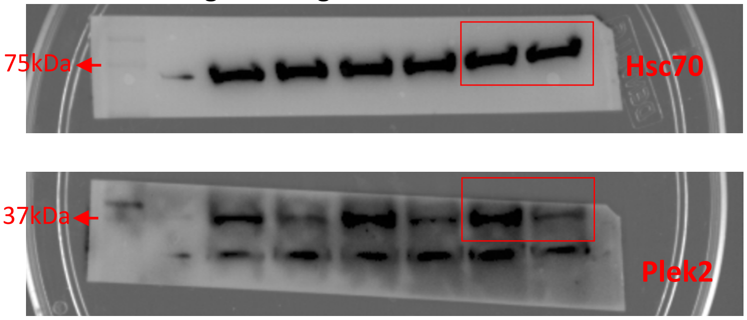

Full unedited gel for Figure 2B

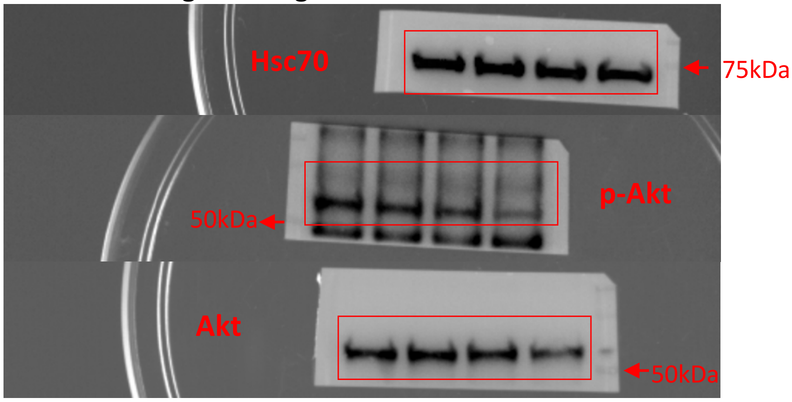

Full unedited gel for Figure 2C

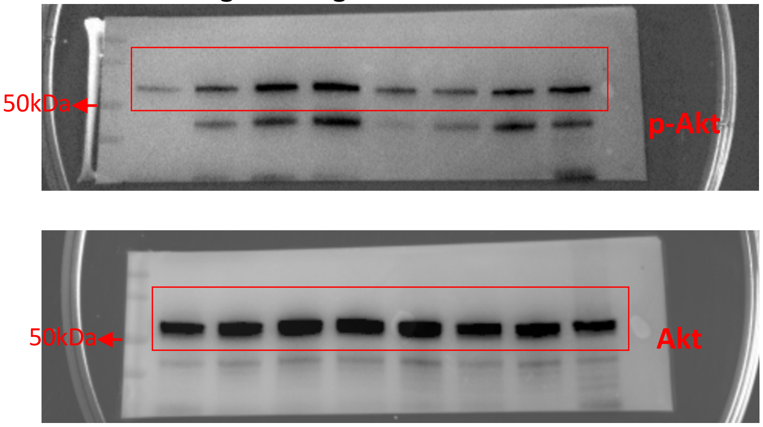

Full unedited gel for Figure 2D

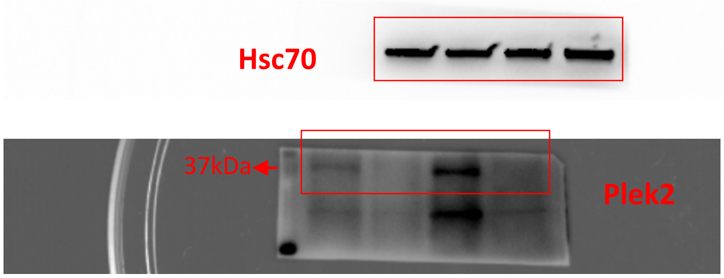

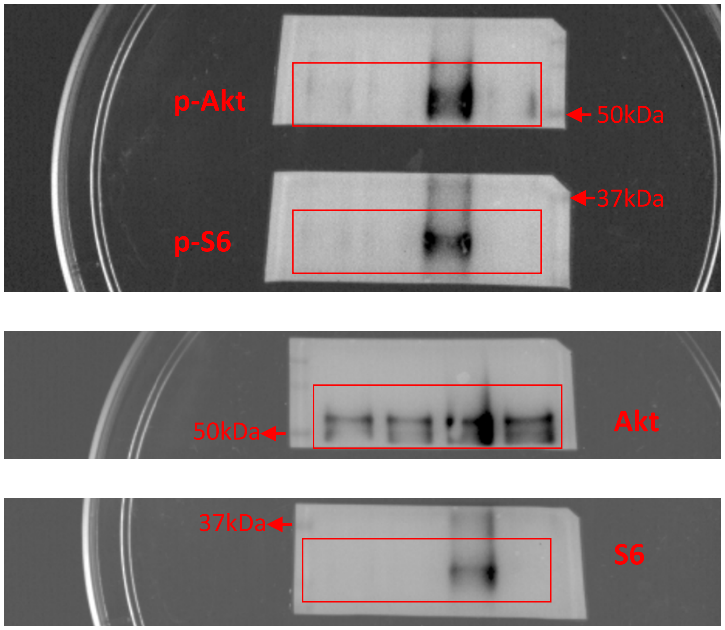

Full unedited gel for Figure 3A

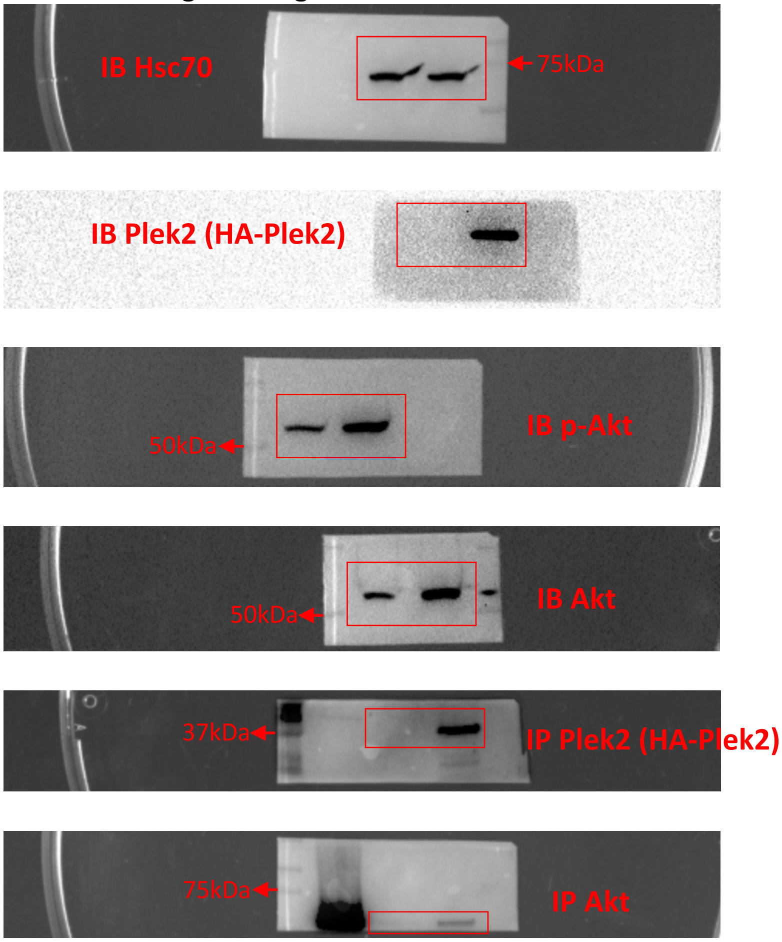

Full unedited gel for Figure 3B

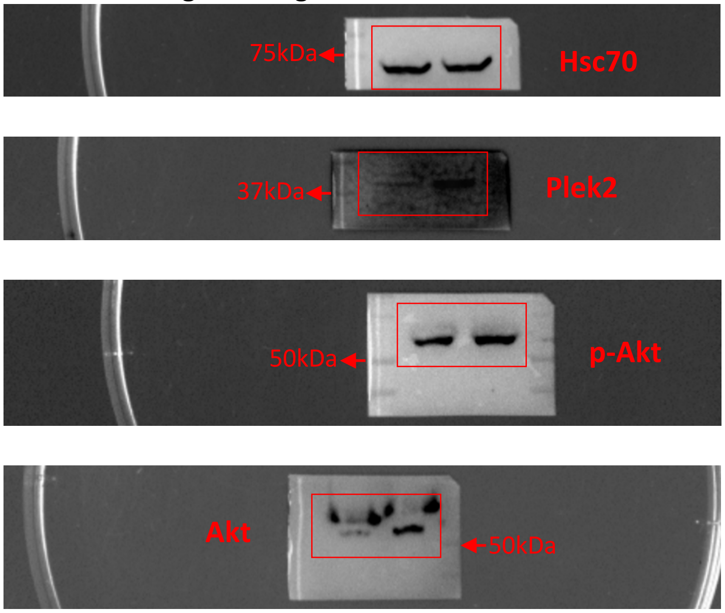

Full unedited gel for Figure 3D

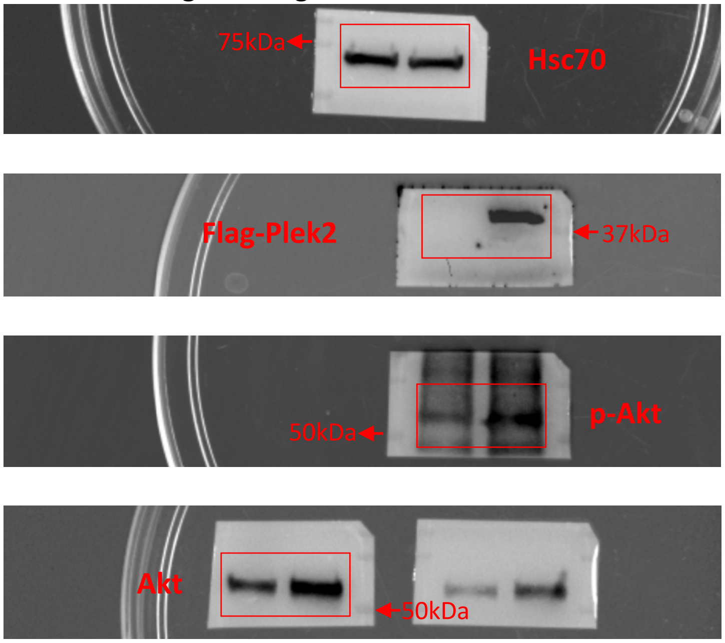

Full unedited gel for Figure 3E

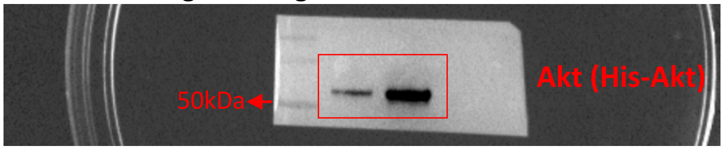

Full unedited gel for Figure 3F

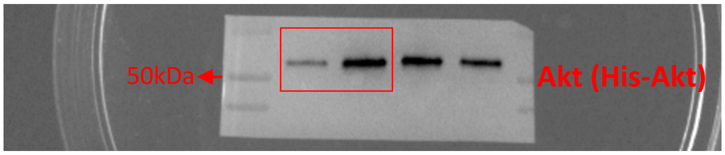

Full unedited gel for Figure 3G

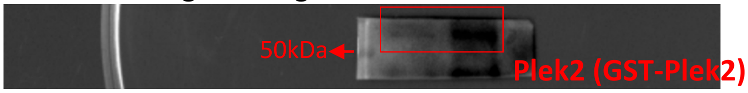

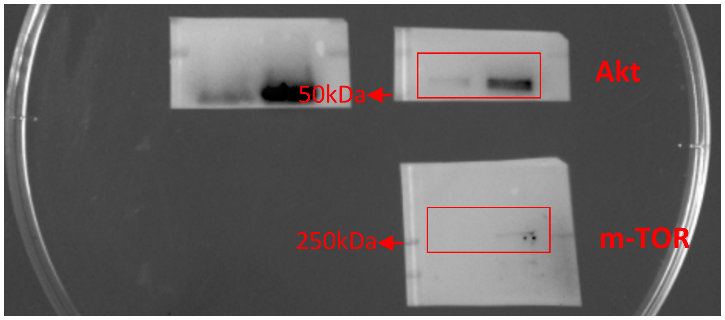

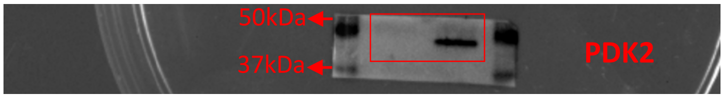

Full unedited gel for Figure 3H

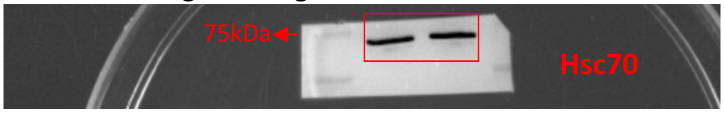

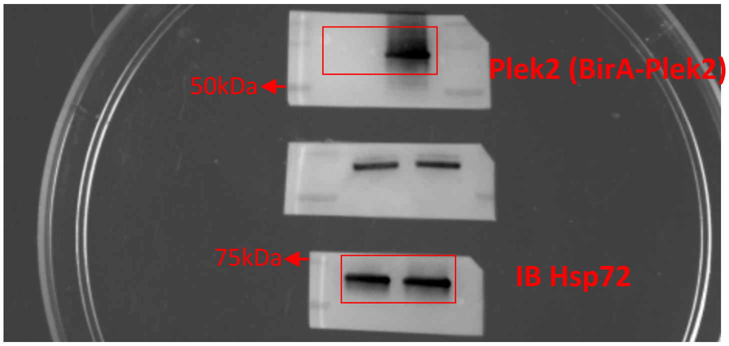

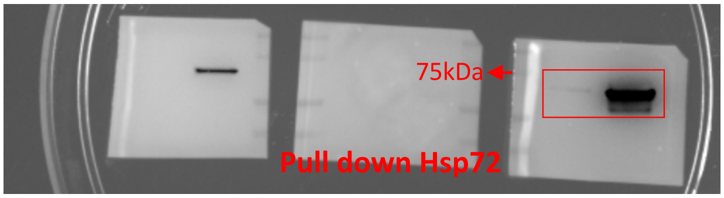

Full unedited gel for Figure 3I

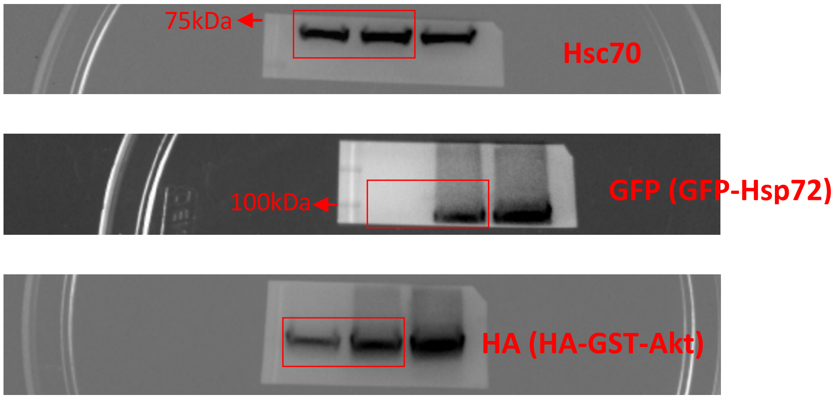

Full unedited gel for Figure 3J

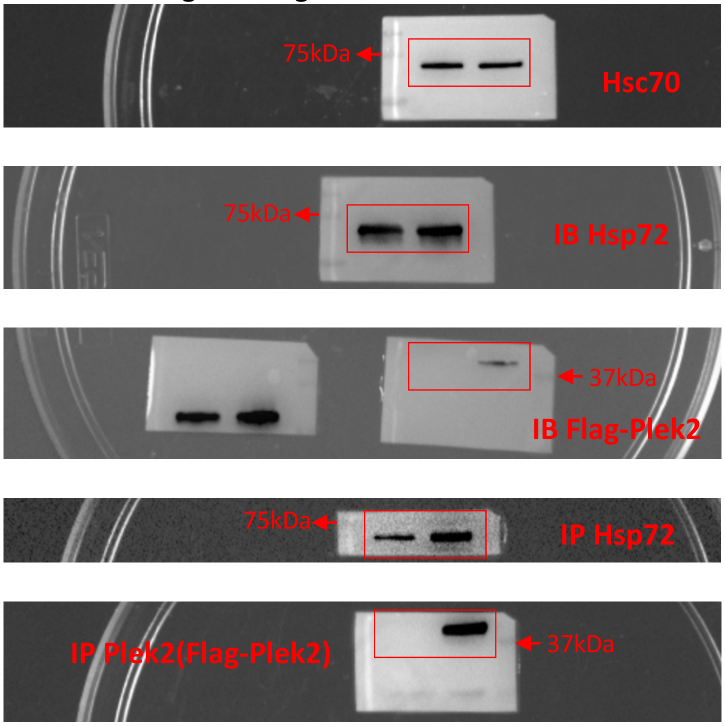

Full unedited gel for Figure 4A

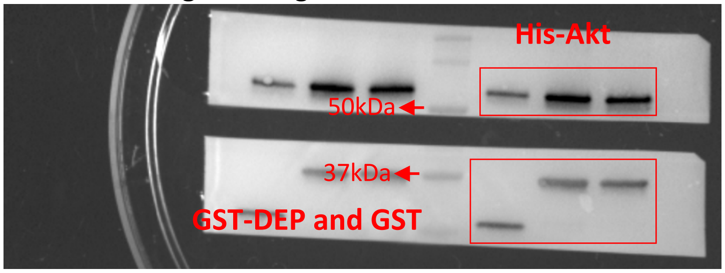

Full unedited gel for Figure 4B

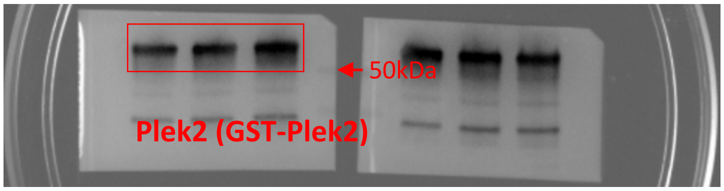

Full unedited gel for Figure 4C

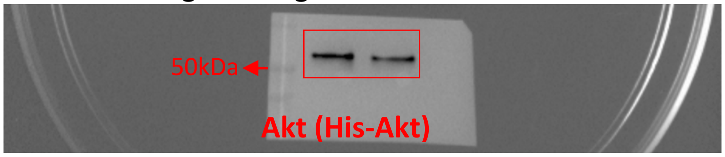

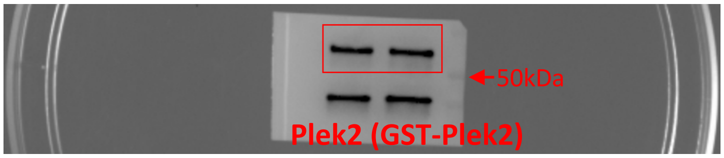

Full unedited gel for Figure 4E

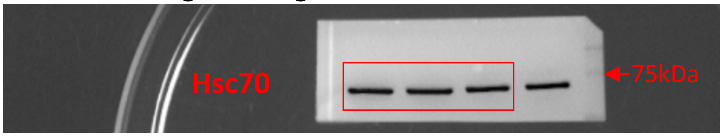

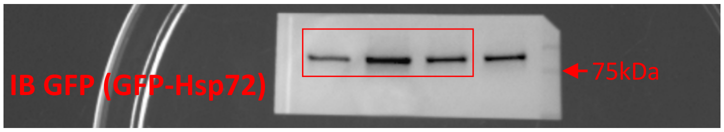

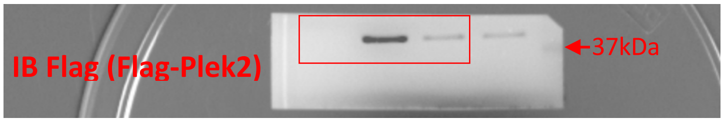

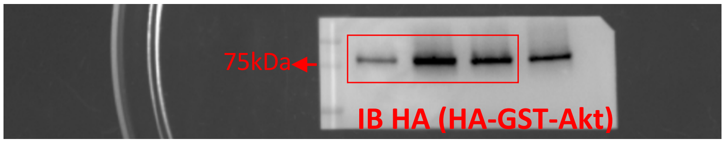

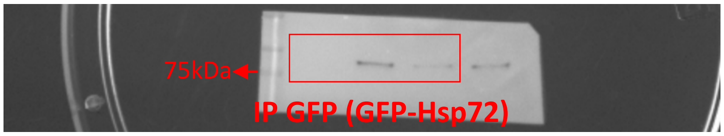

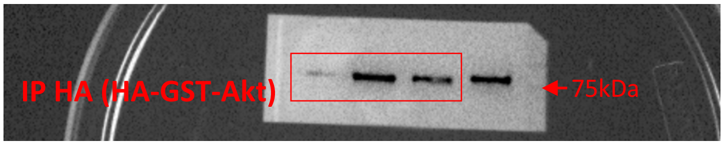

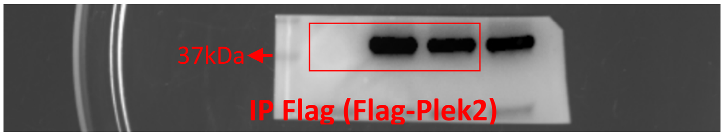

Full unedited gel for Figure 5J

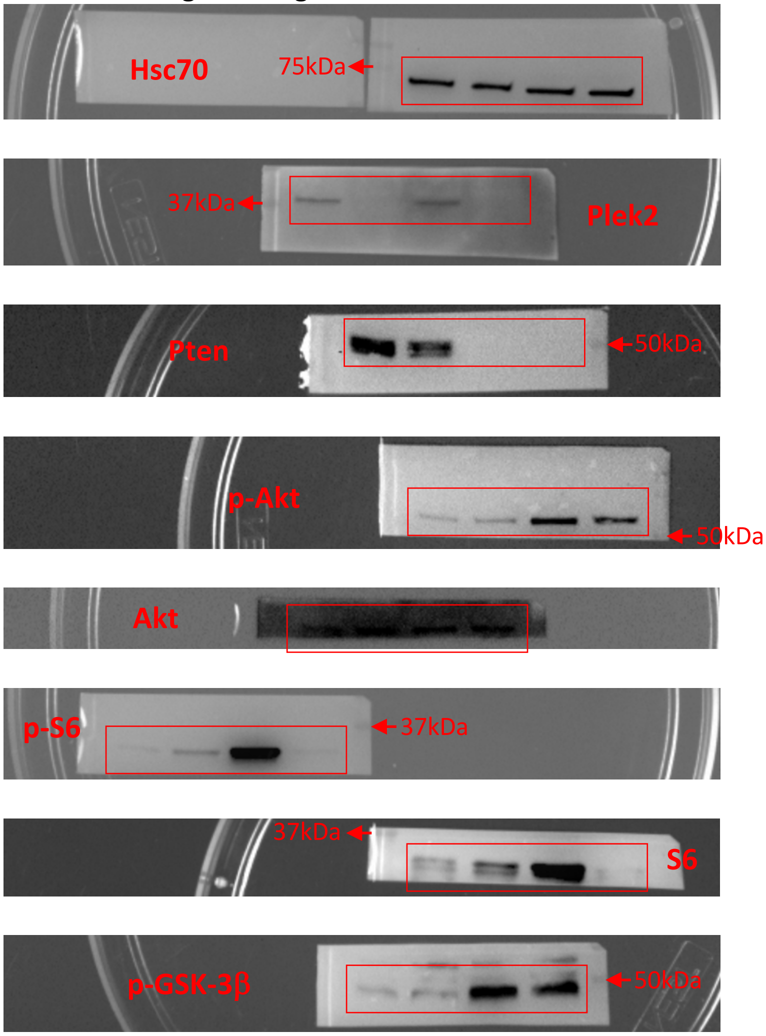

Full unedited gel for Figure 6E

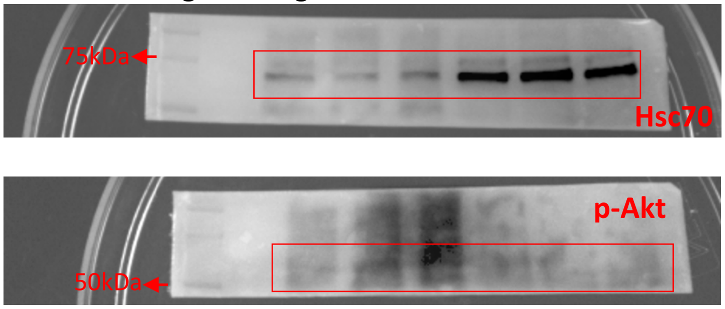

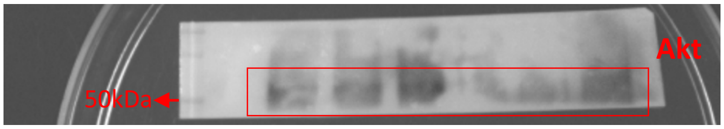

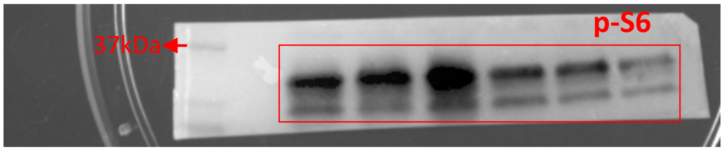

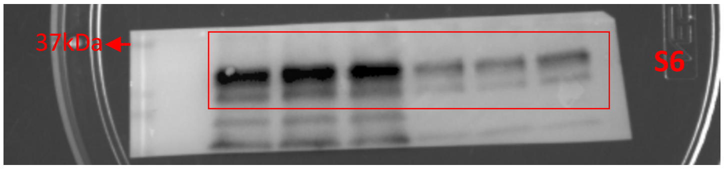

Full unedited gel for Supplemental Figure 1D

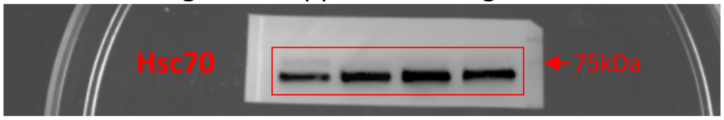

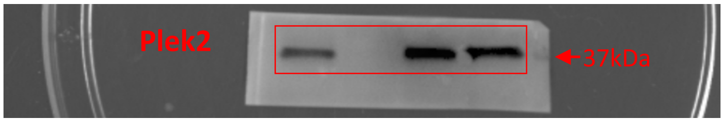

Full unedited gel for Supplemental Figure 3B

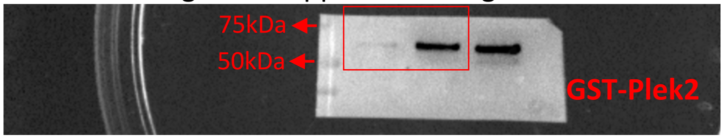

Full unedited gel for Supplemental Figure 3C

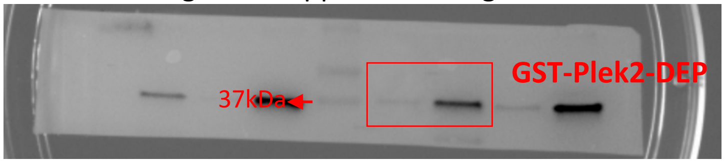

Full unedited gel for Supplemental Figure 3D

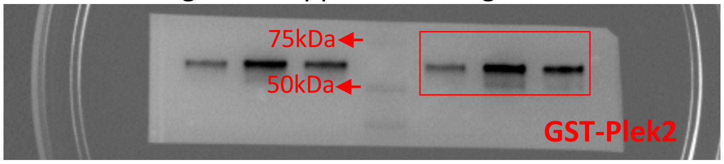

Full unedited gel for Supplemental Figure 3E

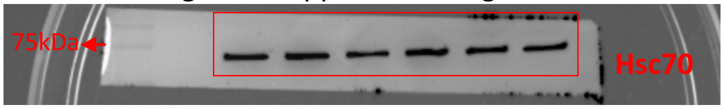

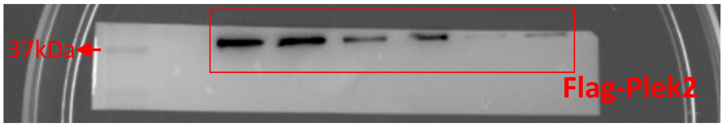

Full unedited gel for Supplemental Figure 5B

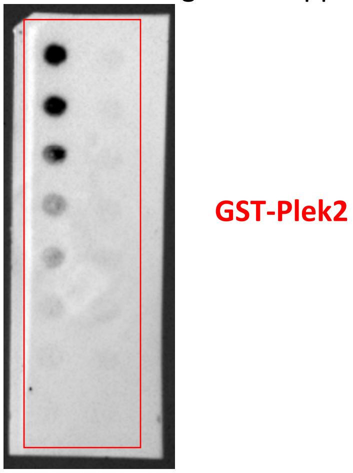

Full unedited gel for Supplemental Figure 5D

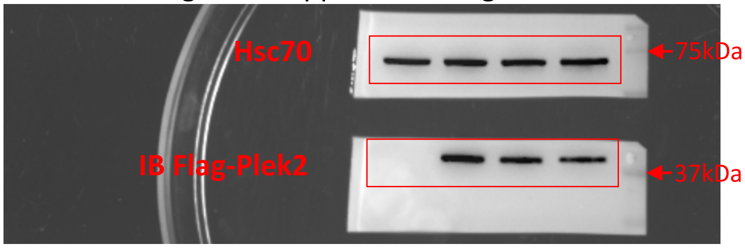

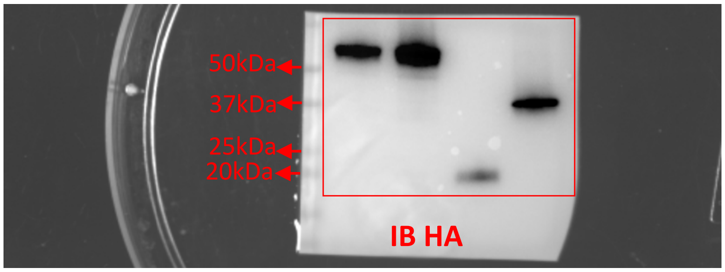

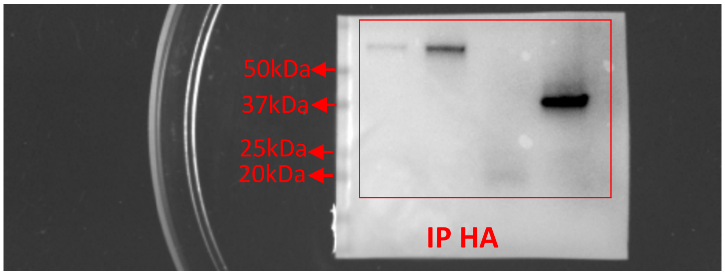

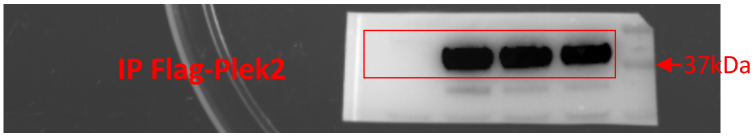

Full unedited gel for Supplemental Figure 5E

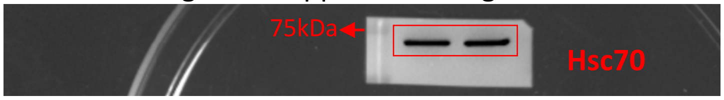

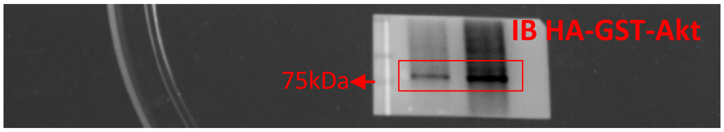

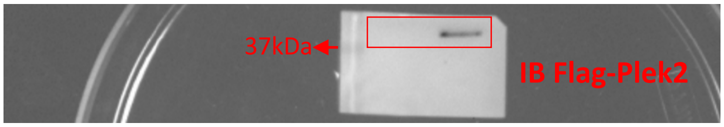

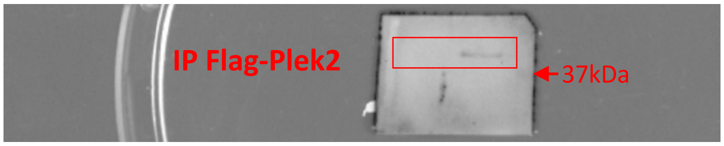

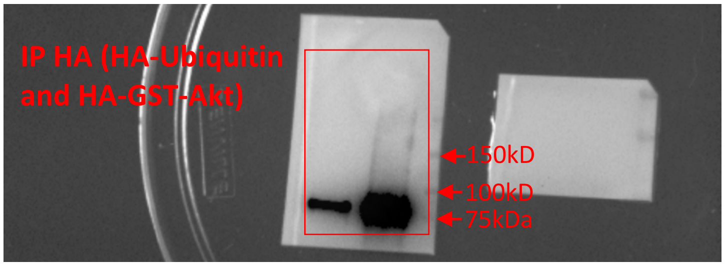

Full unedited gel for Supplemental Figure 5F

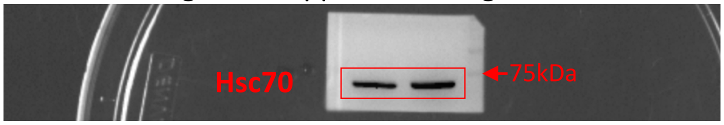

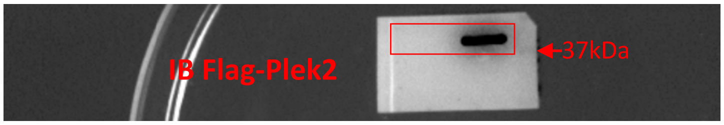

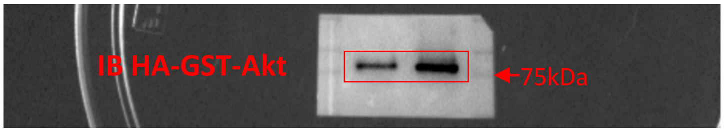

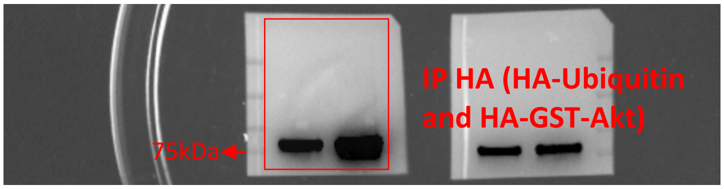

Full unedited gel for Supplemental Figure 5G

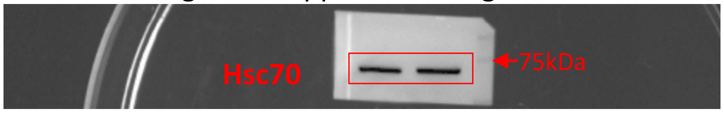

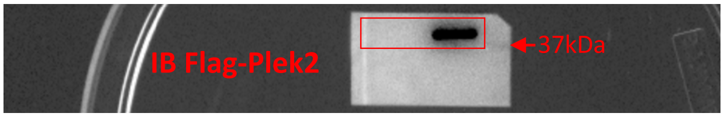

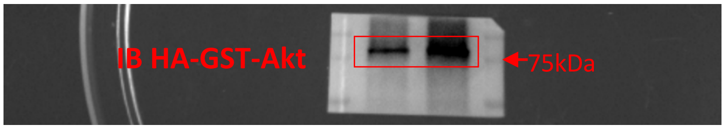

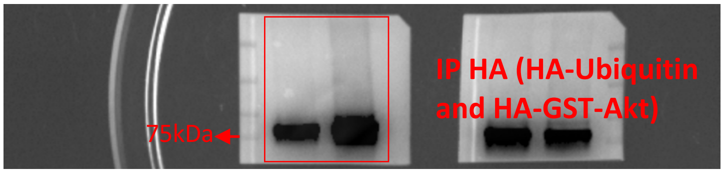

Supplement: Supplemental data [file jci-133-159638-s146.pdf]
